# In-hospital glycemic variability and all-cause mortality among patients hospitalized for acute heart failure

**DOI:** 10.1186/s12933-022-01720-4

**Published:** 2022-12-27

**Authors:** Kyeong-Hyeon Chun, Jaewon Oh, Chan Joo Lee, Jin Joo Park, Sang Eun Lee, Min-Seok Kim, Hyun-Jai Cho, Jin-Oh Choi, Hae-Young Lee, Kyung-Kuk Hwang, Kye Hun Kim, Byung-Su Yoo, Dong-Ju Choi, Sang Hong Baek, Eun-Seok Jeon, Jae-Joong Kim, Myeong-Chan Cho, Shung Chull Chae, Byung-Hee Oh, Seok-Min Kang

**Affiliations:** 1 Division of Cardiology, National Health Insurance Service Ilsan Hospital, Goyang, Korea; 2 Cardiology Division, Department of Internal Medicine, Severance Hospital, Cardiovascular Research Institute, Yonsei University College of Medicine, 50-1, Yonsei-Ro, Seodaemun-Gu, Seoul, 03722 Korea; 3 Division of Cardiology, Seoul National University Bundang Hospital, Seongnam, Korea; 4 Division of Cardiology, Asan Medical Center, Seoul, Korea; 5 Department of Internal Medicine, Seoul National University Hospital, Seoul, Korea; 6 Department of Internal Medicine, Sungkyunkwan University College of Medicine, Seoul, Korea; 7 Department of Internal Medicine, Chungbuk National University College of Medicine, Cheongju, Korea; 8 Department of Internal Medicine, Chonnam National University, Gwangju, Korea; 9 Department of Internal Medicine, Yonsei University Wonju College of Medicine, Wonju, Korea; 10 Department of Internal Medicine, The Catholic University of Korea, Seoul, Korea; 11 Department of Internal Medicine, Kyungpook National University College of Medicine, Daegu, Korea; 12 Department of Internal Medicine, Mediplex Sejong Hospital, Incheon, Republic of Korea

**Keywords:** Glucose metabolism disorder, Glycemic variability, Type 2 diabetes mellitus, Heart failure, Mortality

## Abstract

**Background:**

High glycemic variability (GV) is a poor prognostic marker in cardiovascular diseases. We aimed to investigate the association of GV with all-cause mortality in patients with acute heart failure (HF).

**Methods:**

The Korean Acute Heart Failure registry enrolled patients hospitalized for acute HF from 2011 to 2014. Blood glucose levels were measured at the time of admission, during hospitalization, and at discharge. We included those who had 3 or more blood glucose measurements in this study. Patients were divided into two groups based on the coefficient of variation (CoV) as an indicator of GV. Among survivors of the index hospitalization, we investigated all-cause mortality at 1 year after discharge.

**Results:**

The study analyzed 2,617 patients (median age, 72 years; median left-ventricular ejection fraction, 36%; 53% male). During the median follow-up period of 11 months, 583 patients died. Kaplan–Meier curve analysis revealed that high GV (CoV > 21%) was associated with lower cumulative survival (log-rank P < 0.001). Multivariate Cox proportional analysis showed that high GV was associated with an increased risk of 1-year (HR 1.56, 95% CI 1.26–1.92) mortality. High GV significantly increased the risk of 1-year mortality in non-diabetic patients (HR 1.93, 95% CI 1.47–2.54) but not in diabetic patients (HR 1.19, 95% CI 0.86–1.65, P for interaction = 0.021).

**Conclusions:**

High in-hospital GV before discharge was associated with all-cause mortality within 1 year, especially in non-diabetic patients with acute HF.

**Supplementary Information:**

The online version contains supplementary material available at 10.1186/s12933-022-01720-4.

## Introduction

Dysregulation of hyperglycemia and glycemic variability (GV), which are glycemic parameters, are associated with an increased risk of mortality in critically ill patients [1–4]. As an indicator of variation in the blood glucose level [5], GV has been studied as a clinically significant predictor in various situations. However, most of these studies investigated patients with sepsis or those in the intensive care unit (ICU); hence, there are few results on the impact of glycemic dysregulation in patients with acute heart failure (HF).

Hyperglycemia has been reported to be associated with poor clinical outcomes in patients with cardiovascular diseases, including acute myocardial infarction [6, 7] and acute HF [8]. Generally, hyperglycemia in diabetic patients is attributable to poor glycemic control. In contrast, hyperglycemia in non-diabetic patients may reflect increased sympathetic nervous system (SNS) activation and stressful state [8] because the SNS is associated with the regulation of glucose level via the inhibition of insulin secretion [9]. Moreover, GV has been reported to be correlated with cardiovascular events [10, 11] and increased risk of microvascular and macrovascular complications and mortality in patients with type 2 diabetes [12]. Therefore, it can be inferred that high GV is also associated with adverse outcomes in patients with acute HF because higher GV means a higher risk of not only hyperglycemia but also hypoglycemia, which is a known poor prognostic factor for HF [13].

In this regard, a pilot study focused on the prognostic impact of early GV in a small number of patients with acute HF, which showed that GV measured within the first 24 h of cardiac care unit admission could be an independent predictor of mortality, whereas mean glucose levels were not predictive [14]. Another recent small study noted that GV can predict the 6-month mortality after acute HF hospitalization in diabetic patients [15]. In the present study, we aimed to investigate the association of GV with all-cause mortality up to 1 year in patients with acute HF with or without type 2 diabetes by analyzing data from a Korean acute HF cohort registry.

### Research design and methods

#### Study design and participants

The Korean Acute Heart Failure (KorAHF) registry is a prospective, multicenter, cohort study designed to provide details on demographic and clinical profiles, current diagnostic approaches, treatments, and short- and long-term outcomes among patients with acute HF in Korea. Detailed information on the study design and main results are available in previous reports (ClinicalTrial.gov NCT01389843) [16–19]. Information on patient characteristics, medical history, symptoms and signs, laboratory and echocardiographic findings, prescribed medications, invasive procedures, and outcomes were collected at admission, discharge, and during follow-up (after 30 days, 3 and 6 months, and annually after 5 years). A total of 5625 patients who were hospitalized for acute HF from 10 tertiary hospitals throughout the country were consecutively enrolled between March 2011 and February 2014.

The primary outcome was the all-cause mortality at 1 year, and the secondary outcomes were the all-cause mortality at 6 months, readmission due to HF aggravation at 1 year, and newly detected atrial fibrillation on electrocardiogram during the index hospitalization. The study protocol was approved by the ethics committee/institutional review board of each hospital. All patients provided written informed consent for participation in the registry. The present study was conducted according to the principles of the Declaration of Helsinki.

Of the 5625 patients enrolled in the KorAHF registry, 269 who died at the index hospitalization and 2739 patients who had less than 3 measurements of random glucose level during the index hospitalization were excluded. Finally, 2617 patients were analyzed in this study.

#### Definition of type 2 diabetes and measurement of glycemic variability

Patients with a history of type 2 diabetes and/or hypoglycemic agent use at the time of the index admission as well as HbA_1c_ level ≥ 6.5% (48 mmol/mol) at baseline were considered as having type 2 diabetes in this study. Blood glucose levels were measured through venous blood sampling at the time of admission, during hospitalization, and at discharge. We analyzed only those who had 3 or more blood glucose measurements in this study to measure glucose variability. During the data collection process, we obtained the minimum and maximum blood glucose levels during hospitalization. Therefore, 3 to 4 blood glucose values were analyzed, including the blood glucose levels at the time of admission and discharge, and minimum/maximum levels during hospitalization. We used the standard deviation (SD) and glucose coefficient of variation (CoV, calculated as the ratio of SD divided by the mean glucose level) of the obtained glucose levels to analyze GV [20–22]. We divided patients into two groups based on their CoV as an indicator of GV and investigated the association of GV on the all-cause mortality in patients with acute HF.

### Statistical analyses

Continuous variables with normal distributions are presented as mean ± SD and were compared using the Student’s t-test or the Mann–Whitney U test when group distributions were skewed. Categorical variables were compared using the Chi-square test. We used the Youden method to obtain the optimal cutoff point for GV, seeking equilibrium between sensitivity and specificity [23]. The cumulative probability of events was analyzed using the Kaplan–Meier method, and the log‐rank test was used to compare the probability of event curves between the groups. We used the cutoff point for GV to divide the study population into two groups and investigated the effect of GV on clinical outcomes. Cox proportional hazard regression was used to determine the independent effect of high GV in the study population on the clinical outcomes. Variables found to be statistically significant (*P* < 0.05) in the univariate analysis and variables that could directly affect blood glucose level (e.g., insulin treatment) were included in the multivariate model, except for variables that were closely related to the other variables. All statistical tests were two-tailed, and a *P* value < 0.05 was considered significant. All analyses were performed using R version 4.0.3 (R Foundation for Statistical Computing, Vienna, Austria) using the “pROC” package and “survival” package.

### Data and resource availability

The data sets generated and analyzed during the current study are available from the corresponding author upon reasonable request.

## Results

### Patient characteristics

The baseline characteristics of the study population are shown in Table 1. In this study, 2,617 patients were enrolled. Additional file 1: Table S1 shows the characteristics of those excluded from the study because of a lack of glucose measurements compared to the study population. Compared to the excluded group, the subjects in the study group were older (72 vs 71 years, *P* = 0.003), had a higher prevalence of type 2 diabetes (42% vs 35%, *P* < 0.001) and chronic kidney disease (CKD) (16% vs 12%, *P* < 0.001), a higher proportion of New York Heart Association (NYHA) class III or IV patients (88% vs 81%, *P* < 0.001), and a longer index hospitalization (10 vs 7 days, *P* < 0.001). Overall, the study population had more unfavorable prognostic factors for HF than the excluded group.

**Table 1:** Baseline characteristics according to the 1-year all-cause mortality

	Event (−) (n = 2,034)	Event (+) (n = 583)	Total (n = 2,617)	P value
Age, years	71 (59–78)	76 (70–82)	72 (61–79)	< 0.001
Male sex, n (%)	1097 (54)	282 (48)	1379 (53)	0.02
BMI, kg/m^2^	23 (21–26)	22 (20–25)	23 (21–25)	< 0.001
*Past medical history, n (%)*				
Hypertension	1161 (57)	396 (68)	1557 (60)	< 0.001
Diabetes	840 (41)	270 (46)	1110 (42)	0.035
CKD	272 (13)	153 (26)	425 (16)	< 0.001
Previous HF admission	633 (31)	244 (42)	877 (34)	< 0.001
Ischemic heart disease	544 (2.7)	193 (33)	737 (28)	0.003
Atrial fibrillation	689 (34)	217 (37)	906 (35)	0.148
ICD	20 (1)	8 (1)	28 (1)	0.564
CRT	14 (1)	6 (1)	20 (1)	0.573
*Physical examination on admission*				
SBP, mmHg	130 (110–151)	126 (106–146)	130 (110–150)	0.01
DBP, mmHg	77 (66–90)	76 (61–88)	77 (65–89)	0.008
Pulse rate, beats/min	92 (76–110)	91 (77–110)	91 (76–110)	0.88
NYHA III/IV, n (%)	1,760 (87)	530 (91)	2,290 (88)	0.006
*Echocardiographic parameters*				
LVEF, % (n = 2542)	37 (27–52)	36 (26–52)	37 (27–52)	0.302
LVEF < 40%, n (%)	1,071 (54)	312 (56)	1,383 (54)	0.357
*Laboratory parameters*				
Serum creatinine, mg/dL	1.1 (0.8–1.5)	1.3 (0.9–2.0)	1.1 (0.8–1.6)	< 0.001
eGFR, mL/min/1.73m^2^	65 (43–87)	48 (29–70)	61 (39–84)	< 0.001
Serum sodium, mEq/L	138 (135–141)	137 (133–140)	138 (135–141)	< 0.001
Hemoglobin, g/dL	12.5 ± 2.3	11.5 ± 2.1	12.2 ± 2.3	< 0.001
BNP, pg/mL (n = 1224)	883 (457–1,642)	1,303 (673–2,667)	963 (500–1859)	< 0.001
NT-proBNP, pg/mL (n = 1263)	4827 (2063–10,954)	9,559 (4,026–25,515)	5313 (2373–13,200)	< 0.001
*Treatments during index hospitalization, n (%)*				
Inotropic use	1820 (90)	541 (93)	2361 (90)	0.022
Mechanical ventilation	451 (22)	105 (18)	556 (21)	0.035
RRT	131 (6)	78 (13)	209 (8)	< 0.001
CRRT	55 (42)	37 (47)	92 (44)	0.533
MCSD	138 (7)	27 (5)	165 (6)	0.074
IABP	94 (5)	24 (4)	118 (5)	0.686
ECMO	70 (3)	5 (1)	75 (3)	0.002
Hospital stay, days	10 (6–18)	12 (7–22)	10 (7–19)	< 0.001
*Medications at discharge, n (%)*				
ACEi/ARBs	1371 (67)	325 (56)	1696 (65)	< 0.001
Loop diuretics	1418 (70)	412 (71)	1830 (70)	0.695
Beta-blockers	1009 (50)	223 (38)	1232 (47)	< 0.001
MRAs	947 (47)	243 (42)	1190 (46)	0.042

Values are median (interquartile range), number (%), or mean ± standard deviation

ACEi/ARB, angiotensin-converting enzyme inhibitor/angiotensin II receptor blocker; BNP, brain natriuretic peptide; CRRT, continuous renal replacement therapy; CRT, cardiac resynchronization therapy; DBP, diastolic blood pressure; ECMO, extracorporeal membrane oxygenation therapy; eGFR, estimated glomerular filtration rate; IABP, intra-aortic balloon pump; ICD, implantable cardioverter-defibrillator; LVEF, left ventricular ejection fraction; MCSD, mechanical circulatory support devices; MRA, mineralocorticoid receptor antagonist; NT-proBNP, N-terminal prohormone of brain natriuretic peptide; RRT, renal replacement therapy; SBP, systolic blood pressure

The median age of the study population was 72 years; 53% were male, 42% had type 2 diabetes, and 60% had hypertension. During the follow-up period, 398 (15.2%) patients died within 6 months, 583 (22.3%) patients died within 1 year, and 576 (22.0%) patients experienced HF readmission (Additional file 1: Table S2). We compared the two patient groups according to the 1-year all-cause mortality (Table 1). Compared to those who survived at 1-year, the patients who died were older (76 vs 71 years, *P* < 0.001) and had a higher prevalence of hypertension (68% vs 57%, *P* < 0.001), type 2 diabetes (46% vs 41%, *P* = 0.035), previous HF admission (42% vs 31%, *P* < 0.001), and CKD (26% vs 13%, *P* < 0.001), and they were treated with renal replacement therapy (13% vs 6%, *P* < 0.001) more frequently.

### Glucose parameters according to 1-year mortality

Additional file 1: Table S3 shows the parameters related to glycemic status according to the 1-year all-cause mortality. Compared to those who survived at 1-year, the patients who died had a significantly higher mean glucose level (133 vs 125 mg/dL, *P* < 0.001), SD of glucose level (42.0 vs 34.6 mg/dL, *P* < 0.001), and CoV of glucose level (31.0% vs 27.5%, *P* < 0.001). Although the prevalence of type 2 diabetes was higher in the patients who died, the HbA_1c_ level was not significantly different between the two groups (6.6% vs 6.4% [49 vs. 46 mmol/mol], *P* = 0.155). There was no significant difference in the rate of oral hypoglycemic agent use, including sulfonylureas, and insulin treatment between the two groups. The distributions of blood glucose levels were shown in Additional file 2: Fig. S1, Additional file 3: Fig. S2, Additional file 4: Fig. S3. Scatter plot of the blood glucose level of all subjects (at the time of admission, minimum, maximum values during hospitalization, and at discharge, respectively) (Additional file 2: Fig. S1) and the distribution of blood glucose levels according to the presence of diabetes (Additional file 3: Fig. S2) or insulin use (Additional file 4: Fig. S3) were presented.

Additional file 5: Fig. S4 shows the spline curves for the 1-year all-cause mortality according to the SD (Additional file 5: Fig. S4A) and CoV of glucose level (Additional file 5: Fig. S4B). These figures show that the higher the SD and CoV, the higher the hazard ratio (HR) of all-cause mortality (P < 0.001, for both). Thereafter, we attempted to obtain the optimal cutoff point of each parameter to maximize the log-rank test statistics for mortality. In the study population, the cutoff value for the SD and CoV were 24.5 mg/dL and 20.9%, respectively (Additional file 1: Table S4). In patients with and without type 2 diabetes, the cutoff value for the SD and CoV were 68.3 mg/dL and 21.8%, and 24.5 mg/dL and 20.5%, respectively. Since the SD cutoff difference between patients with and without type 2 diabetes was larger, we decided to use the CoV as an indicator of GV instead of the SD and divided the study population into two groups based on the CoV. Thereafter, the cutoff value was set as 21%, which was the most approximate integer value obtained from the total study group and the type 2 diabetes/non-diabetes subgroups.

### Clinical outcomes and glucose variability parameters

Then we analyzed the glycemic parameters and clinical events of the study population according to GV (cutoff CoV = 21%). The mean glucose level, type 2 diabetes prevalence, and HbA_1c_ values were significantly higher (6.6% vs 6.2%, *P* < 0.001) in the high GV group (CoV > 21%, n = 1693) than in the low GV group (CoV ≤ 21%, n = 924) (Table 2). Regarding clinical events, 1-year all-cause mortality was more prevalent in the high GV group than in the low GV group, but HF readmission was not significantly different between the two groups. In terms of arrhythmic events regarding the GV, among the patients without previous history of atrial fibrillation at baseline (n = 1711), patients with the high GV were more frequently detected to have new atrial fibrillation on the electrocardiogram during the index hospitalization compared to those with the low GV (13% vs. 6%, *P* = 0.02). Other clinical characteristics (Additional file 1: Table S5) showed that the high GV group had a higher prevalence of patients with CKD (19.0% vs 11.1%, *P* < 0.001) and ischemic heart disease (30.0% vs 24.8%, *P* = 0.005), higher proportion of NYHA III or IV patients (88.5% vs 85.6%, *P* = 0.035), and higher in-hospital treatment rate with mechanical ventilation (28.9% vs 7.3%, *P* < 0.001) and mechanical circulatory support devices (8.0% vs 3.2%, *P* < 0.001), suggesting that the high GV group had a more severe clinical course of HF.

**Table 2:** Glucose measurements, treatment for type 2 diabetes, and clinical events during the follow-up period according to glycemic variability

	Low GV (CoV ≤ 21%) n = 924	High GV (CoV > 21%) n = 1,693	P value
*Glucose parameters*			
Mean glucose level, mg/dL	109 (97–125)	142 (118–177)	< 0.001
Difference of glucose level^*^, mg/dL	27 (15–39)	115 (75–180)	< 0.001
SD of glucose level, mg/dL	13.3 (8.0–19.3)	55.8 (36.9–85.7)	< 0.001
CoV of glucose level, %	12.2 (7.5–16.8)	38.4 (29.2–51.9)	< 0.001
DM prevalence, n (%)	241 (26)	869 (51)	< 0.001
HbA1c, % [mmol/mol]	6.2 (5.8–6.8) [44 (40–51)]	6.6 (5.9–7.5) [49 (41–58)]	< 0.001
*Treatment for type 2 diabetes, n (%)*			
Insulin treatment during hospitalization	116 (13)	689 (41)	< 0.001
Oral hypoglycemic agent use on admission	113 (12)	395 (23)	< 0.001
Sulfonylurea	68 (7)	331 (20)	< 0.001
Metformin	88 (10)	300 (18)	< 0.001
Thiazolidinedione	5 (1)	17 (1)	0.310
Alpha glucosidase inhibitor	22 (2)	65 (4)	0.061
DPP4 inhibitor	27 (3)	111 (7)	< 0.001
*Clinical events, n (%)*			
HF readmission	191 (21)	385 (23)	0.241
6-month all-cause mortality	87 (9)	311 (18)	< 0.001
1-year all-cause mortality	152 (17)	431 (26)	< 0.001
Newly detected AF during hospitalization (n = 1711)^†^	49 (9%)	149 (13%)	0.020

Values are median (interquartile range) or number (%)

CoV, coefficient of variation; DPP-4, dipeptidyl peptidase-4; SD, standard deviation. Other abbreviations as in Table 1

^*^Difference of glucose level: maximal value – minimal value of measured glucose level

^†^Events were evaluated among the participants who did not have previous history of atrial fibrillation at the time of admission

**Fig. 1:**
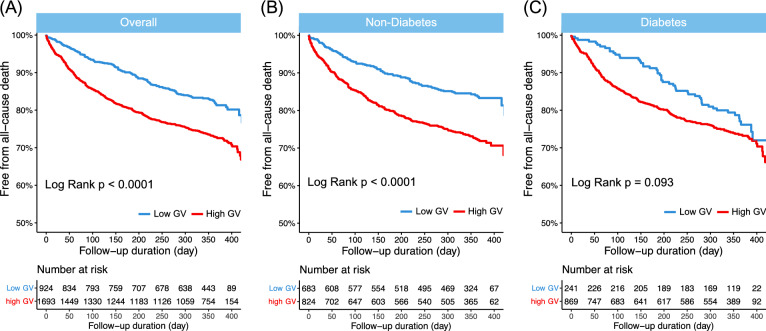
In-hospital glycemic variability and all-cause mortality among patients hospitalized for acute heart failure. Kaplan–Meier curves for the 1-year all-cause mortality according to GV. High GV was defined as CoV > 21%, and low GV was defined as CoV ≤ 21%. For total population and non-diabetes, high GV was associated with significant higher risk of all-cause death. CoV, coefficient of variation; GV, glycemic variability

**Fig. 2:**
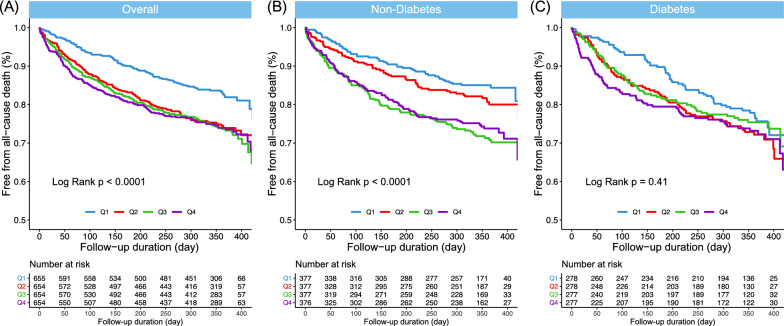
Kaplan–Meier curves for the 1-year all-cause mortality according to quartiles (Q1-4) of glucose variability. The range of coefficient of variation (CoV) in the quartiles was < 16.1; 16.1–28.5; 28.5–43.9; > 43.9 (%) in the study population; < 13.2; 13.2–22.8; 22.8–35.4; > 35.5 (%) in the non-diabetes group; and < 23.5; 23.5–37.5; 37.5–51.9; > 51.9 (%) in the type 2 diabetes group. Q1: quartile 1; Q2: quartile 2; Q3: quartile 3; Q4: quartile 4. P-values were calculated using log-rank statistics

We evaluated the association of GV with the 1-year all-cause mortality via Kaplan–Meier survival analysis. Among the total study population, the high GV group had a higher risk of all-cause mortality (log-rank *P* < 0.001) than the low GV group (Fig. 1). When analyzing the study population according to diabetic status, this association was still significant at both 6-month and 1-year follow-up in non-diabetic patients (log-rank *P* < 0.001, both). However, in diabetic patients, the association was significant at 6-month follow-up period (log-rank *P* = 0.00024), but this association was attenuated at 1-year follow-up (log-rank *P* = 0.093). When analyzing by quartile, we determined that the higher the GV, the higher the risk of 1-year all-cause mortality among all study subjects and in patients without type 2 diabetes (Fig. 2). Baseline characteristics by dividing dichotomized GV groups and diabetes is shown in Additional file 1: Table S6.

Based on these results, Cox proportional-hazard regression was used to determine the association of high GV with the 1-year all-cause mortality. In the univariate analysis (Additional file 1: Table S7), older age, male sex, low body mass index, low systolic and diastolic blood pressure, low estimated glomerular filtration rate, high natriuretic peptide levels (brain natriuretic peptide ≥ 500 pg/ml or N-terminal prohormone of brain natriuretic peptide ≥ 1000 pg/ml) at admission, hypertension, CKD, previous HF admission, ischemic heart disease, and high GV (CoV > 21%) were significant predictors of all-cause mortality. Also, inotropic therapy, renal replacement therapy, mechanical circulatory support device therapy during hospitalization, and longer duration of hospitalization were associated with increased risk for all-cause mortality.

When all these significant variables were integrated into multivariate Cox regression analysis (Table 3), high GV still remained a significant predictor of 1-year all-cause mortality (HR 1.59, 95% CI 1.29–1.96, *P* < 0.001), and this association was also significant in the 6-month all-cause mortality (HR 1.97, 95% CI 1.50–2.57, *P* < 0.001) (Table 4). In addition, we analyzed the Cox proportional-hazard regression according to diabetic status. In non-diabetic patients, high GV was significantly associated with both 6-month (HR 2.00, 95% CI 1.44–2.78, *P* < 0.001) and 1-year all-cause mortality (HR 1.92, 95% CI, 1.47–2.51, *P* < 0.001). In diabetic patients, however, high GV was only associated with 6-month all-cause mortality (HR 2.04, 95% CI, 1.26–3.30, *P* = 0.004), but not with 1-year all-cause mortality (HR 1.23, 95% CI, 0.89–1.70, *P* = 0.215) (Table 4).

**Table 3:** Multivariate Cox regression analysis for the 1-year all-cause mortality

	Hazard ratio (95% confidence interval)	P value
Age (per 1 year increase)	1.039 (1.030–1.048)	< 0.001
Male (vs. female)	1.030 (0.854–1.241)	0.738
BMI (per 1 kg/m^2^ increase)	0.941 (0.915–0.967)	< 0.001
SBP (per 1 mmHg increase)	0.993 (0.989–0.997)	0.002
DBP (per 1 mmHg increase)	1.005 (0.998–1.012)	0.190
Hemoglobin (per 1 g/dL increase)	0.928 (0.886–0.971)	0.001
eGFR (per 1 mL/min/1.73m^2^ increase)	0.996 (0.992–1.000)	0.054
High NP level (yes vs. no)^*^ (n = 2391)	1.454 (1.065–1.985)	0.018
LVEF (per 1% increase) (n = 2542)	0.993 (0.987–0.999)	0.046
*Past medical history*		
Hypertension (yes vs. no)	1.231 (0.999–1.517)	0.052
Type 2 diabetes (yes vs. no)	1.019 (0.821–1.264)	0.865
CKD (yes vs. no)	1.221 (0.943–1.583)	0.130
Previous HF admission (yes vs. no)	1.107 (0.914–1.341)	0.297
Ischemic heart disease (yes vs. no)	0.926 (0.756–1.133)	0.454
*Treatments during index hospitalization*		
Inotropic use (yes vs. no)	1.045 (0.740–1.476)	0.802
RRT (yes vs. no)	1.662 (1.219–2.266)	0.001
Insulin treatment during hospitalization (yes vs. no)	0.888 (0.710–1.111)	0.299
Duration of hospitalization (per 1 day increase)	1.003 (0.998–1.007)	0.238
*Medications at discharge*		
ACEi/ARBs (yes vs. no)	0.684 (0.566–0.826)	< 0.001
Beta-blockers (yes vs. no)	0.752 (0.625–0.904)	0.002
MRAs (yes vs. no)	1.054 (0.872–1.274)	0.300
*Glucose parameters during hospitalization*		
CoV > 21% (yes vs. no)	1.558 (1.262–1.923)	< 0.001

HRs are expressed as “vs” for dichotomous variables and per unit for continuous variables. Men coded as 1 and women as 0. Yes coded as 1 and no as 0

HR of each variable was analyzed after adjustment for age, sex, BMI, SBP, DBP, hypertension, type 2 diabetes, CKD, previous HF admission history, ischemic heart disease, LVEF on echocardiography, baseline hemoglobin and eGFR level, high natriuretic peptide levels at admission, inotropic use, renal replacement therapy, insulin treatment during hospitalization, duration of hospitalization, and use of ACEi/ARB, beta-blocker, and mineralocorticoid receptor antagonist at discharge

Abbreviations as in Tables 1 and 2

^*^Data analyzed with high natriuretic peptide (BNP ≥ 500 pg/ml or NT-proBNP ≥ 1000 pg/ml) levels at admission, in available subjects (n = 2391)

**Table 4:** Multivariate Cox regression analysis of glycemic variability (CoV > 21%) for the 6-month and 1-year all-cause mortality

	Total population		Non-diabetes		Type 2 diabetes	
	HR (95% CI)	P value	HR (95% CI)	P value	HR (95% CI)	P value
6-month all-cause mortality	1.92 (1.47–2.52)	< 0.001	2.06 (1.47–2.87)	< 0.001	1.94 (1.20–3.15)	0.007
1-year all-cause mortality	1.56 (1.26–1.92)	< 0.001	1.93 (1.47–2.54)	< 0.001	1.19 (0.86–1.65)	0.296

HR was analyzed after the same adjustment for the relevant variables in Table 3

CoV, coefficient of variation

**Fig. 3:**
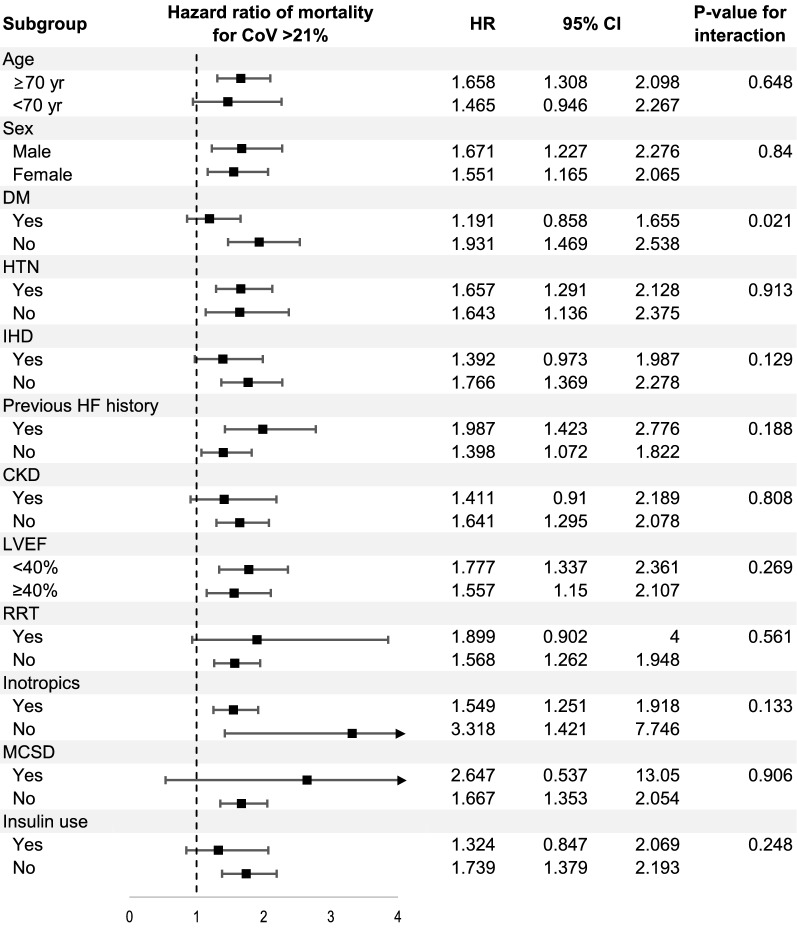
Subgroup analysis for risk of the 1-year all-cause mortality according to glycemic variability (cutoff CoV = 21%). CKD, chronic kidney disease; CoV, coefficient of variation; DM, type 2 diabetes mellitus; HF, heart failure; HR, hazard ratio; HTN, hypertension; IHD, ischemic heart disease; LVEF, left ventricular ejection fraction; MCSD, mechanical circulatory support devices; RRT, renal replacement therapy

Figure 3 illustrates the subgroup analysis based on the classification of each clinical component. Regarding the association of high GV and 1-year all-cause mortality, we found a significant difference between the subgroups with and without type 2 diabetes (*P* for interaction = 0.018) as shown above. In other subgroups, including those who used medications such as renin–angiotensin–aldosterone inhibitors, beta-blockers, mineralocorticoid receptor antagonists, loop diuretics, or sulfonylureas, there was no significant interaction.

## Discussion

The principal finding of the current study is that a high GV before discharge was associated with a higher risk of all-cause mortality compared to a low GV in hospitalized patients with acute HF. Since our study was a retrospective analysis, we tried to adjust clinical variables to present that the GV was independently associated with mortality. Several baseline demographics and clinically relevant variables were analyzed in the multivariate analysis models. For example, although the frequency of insulin use was higher in patients with high GV, it was not found to be associated with the risk of mortality in the univariate analysis. However, because insulin therapy is known to be associated with hypoglycemic events and adverse outcomes [24, 25], the use of insulin was also adjusted to the multivariate models. In addition, since the longer hospital stay may reflect the high severity of heart failure and have more chances to be treated with intravenous infusion containing glucose, so the analysis was adjusted for the duration of the index hospitalization. After the adjustments, the result showed that a high GV was associated with higher mortality, especially in non-diabetic patients with acute HF.

### Glycemic variability in cardiovascular diseases

Many studies have indicated that dysregulation of glucose levels is associated with mortality. Most of these studies included patients with acute or severe conditions such as those in the ICU [1–3], and some reports showed its association with cardiovascular events in patients with ischemic heart disease [10, 11]. Another large-scale prospective cohort study which analyzed more than 4,800 participants with or without type 2 diabetes, a post hoc analysis of data from the Antihypertensive and Lipid-Lowering Treatment to Prevent Heart Attack Trial (ALLHAT), demonstrated that visit-to-visit GV was associated with all-cause mortality [26]. A recent meta-analysis by Chen et al. showed that long-term GV leads to a higher risk of mortality and various diabetes-related outcomes in patients with diabetes [27]. However, there have been few studies on the impact of GV in acute HF.

Dungan et al. investigated 748 hospitalized patients with HF and revealed that the GV index could predict in-hospital mortality [28]. However, this study focused only on short-term (< 60 days) outcomes, whereas the present study analyzed the 1-year mortality outcomes. Lazzeri et al. later observed that the GV on the first day of admission in the ICU was associated with mortality in 247 patients with acute HF [14]. They revealed that GV was an independent predictor of mortality within a median follow-up period of 10 months, but the mean glucose levels were not. They used two methods to measure GV: the SD of glucose levels and the mean absolute glucose change per hour. However, the number of participants was small, and the study focused on acute coronary syndrome (72% of total study population). In our study, we analyzed patients with ischemic (28%) and non-ischemic (72%) HF, and subgroup analysis showed consistent results regardless of the presence of either ischemic heart disease or HF with reduced ejection fraction (≤ 40%). In addition, a recent study showed that elevated GV in patients with acute HF with type 2 diabetes (n = 214) predicted the 6-month mortality (HR 2.34, *P* = 0.03) [15]. However, the authors only focused on diabetic patients, and the study population was small.

In addition, we showed that atrial fibrillation was more commonly detected during hospitalization in patients with high GV than those with low GV among the patients without previously reported AF. This finding is in line with the previous studies that revealed cardiac arrhythmias were common in insulin-treated patients with type 2 diabetes and are associated with GV [29], which was also independently associated with the development of new-onset atrial fibrillation in these diabetic patients [30].

### Possible mechanisms for the association between glycemic variability and mortality in HF

Glycemic dysregulation might reflect poor pancreatic β-cell function and the inadequate release of endogenous insulin; therefore, hyperglycemia and high GV would be more pronounced in patients with type 2 diabetes with worse glucose control and longer disease duration [15]. However, in acute HF, SNS activation is one of the important neurohormonal mechanisms related to disease progression [31]. Thus, high GV may reflect an acute stressful state and increased sympathetic tone even in non-diabetic patients with acute HF. This may mean that the HR of high GV for mortality at 6 months may be higher than that at 1 year, which is consistent with our findings (Table 4). The use of HF medications, such as renin–angiotensin–aldosterone inhibitors, beta-blockers, mineralocorticoid receptor antagonists, or loop diuretics, may affect the neurohormonal system. However, the present results showed that the effect of GV on mortality was significant regardless of the use of these drugs.

There may be several explanations that high GV is associated with higher mortality in acute HF patients, particularly non-diabetic patients. In acute HF patients, there is a period wherein the readmission rate is high early after discharge, which is often referred to as a “vulnerable phase” because it determines the prognosis in post-discharge period [32]. The present results showed that a high GV during hospitalization was associated with early mortality within 6 months and 1 year after discharge. This is in line with another study of the KorAHF registry that showed an association between hyperglycemia and a high 1-year mortality rate in non-diabetic patients [8]. It may be assumed that GV could be affected by SNS activation followed by decompensation rather than glucose metabolism itself in non-diabetic patients. Also, a previous study suggested that oscillating glucose levels can have more deleterious effects than constant high glucose on endothelial function and oxidative stress in both subjects with normoglycemia and with type 2 diabetes [33]. A recent study showed that greater short-term GV was associated with increased risk for all-cause mortality even in type 2 diabetic patients with well-controlled glucose profile by continuous glucose monitoring [34]. Moreover, in another recent study that analyzed echocardiographic parameters in non-diabetic subjects, visit-to-visit GV was also associated with left ventricular diastolic dysfunction, also supporting our findings [35]. In the above-mentioned post hoc analysis, the results suggested that greater visit-to-visit GV is associated with an increased risk for all-cause mortality, especially among people without type 2 diabetes [26]. These previous results support our findings that GV has a significant association with mortality even in patients without diabetes. Although the measurement of GV in our study was short-term and an in-hospital parameter, which is different from the visit-to-visit GV, we showed that high GV was associated with higher mortality in non-diabetic patients. Even in patients with type 2 diabetes, our results showed that high GV was associated with higher mortality only at 6 months, not 1-year after discharge, which can also be considered the effect of acute stress in the post-discharge period of acute HF.

In the present study, the mean glucose level, prevalence of type 2 diabetes, and use of exogenous insulin treatment were higher in the high GV group than in the low GV group. It can be expected that insulin treatment and GV are closely related because hyperglycemia requires insulin treatment, which may cause hypoglycemic side effects. In a study of the KorAHF registry patients with type 2 diabetes, the mortality rate was higher in patients treated with insulin than in patients treated with oral hypoglycemic agents alone [25]. However, our study analyzed patients with and without type 2 diabetes, and we found that insulin use was not significantly associated with mortality in the Cox analysis. There may be two reasons for this difference. First, the previous study was a result of comparing insulin treatment and oral hypoglycemic agents in diabetic patients; and second, we enrolled subjects whose glucose levels were measured at least three times during hospitalization, and patients who died during hospitalization were excluded from our study population.

A recent randomized clinical trial showed that several indicators, including HbA_1c_ level, were more significantly improved when using continuous glucose monitoring compared to conventional glucose measurement in diabetic patients receiving insulin treatment [36]. In acute HF patients, the more severe the HF, the higher the GV tends to be due to the influence of increased neurohormonal activity or inflammation with deteriorated hemodynamic conditions. However, this does not mean that any interventions to reduce GV improve the outcomes of acute HF patients, so further research is needed to determine whether GV may be a therapeutic target in this population. Considering the increasing use of wearable devices and telemonitoring in clinical practice, it is expected that more studies will be warranted to reveal the clinical usefulness of GV monitoring using continuous glucose monitoring in outpatient care and impact of intervening to reduce GV in HF patients.

## Study limitations

A major strength of our study is it is a prospective multicenter cohort study of patients with acute HF. However, several limitations also are noted. First, there was no pre-specified method for glucose measurements during the hospital stay in this cohort study. Accordingly, only subjects who had blood glucose measurements 3- or 4-times during hospitalization were included in the study, which may have a selection bias. This bias could have been improved and more accurate results for glucose variability could be obtained if a prospectively planned and repeated blood glucose data collection was performed. In addition, continuous glucose monitoring was not performed either. While it was not possible to measure glucose levels in a predefined manner, the advantage of this study is that it was performed by analyzing the maximum and minimum values of the measured glucose levels, and we obtained GV values in a large population of patients with acute HF regardless of diabetic status. Second, because it was difficult to investigate the causes of deaths of the study population, our study is the result of analyzing all-cause mortality, not cardiovascular outcome. Although cardiac death, including sudden cardiac death, is the leading cause of death in acute HF patients, we could not capture all data regarding deaths. Because SNS activity plays a major role in the pathophysiology of sudden cardiac death [37], HF [31], and GV [9], it is one of the limitations of our study. Third, data on the use of sodium-glucose cotransporter 2 inhibitors were not available. They may have provided additional insight for the interaction between GV and sodium-glucose cotransporter 2 inhibitors on clinical outcomes because sodium-glucose cotransporter 2 inhibitor is a novel treatment option for improving clinical outcomes in patients with HF with reduced and preserved ejection fraction [38, 39]. In addition, the effect of HF medications, such as diuretics (e.g., thiazides, acetazolamide rather than loop diuretics), that may also affect the SNS and renin–angiotensin–aldosterone system were not analyzed in detail. Despite these limitations, our study highlights the powerful association between high GV and risk of all-cause mortality in acute HF patients.

## Conclusions

We found that high GV in hospitalized patients with acute HF was an independent predictor of all-cause mortality within 1 year, especially in non-diabetic patients. Further studies are needed to assess whether efforts to modify GV are beneficial for reducing mortality in patients with acute HF.

## Supplementary Information

**Additional file 1: **Additional Tables.

**Additional file 2: Fig. S1.** Scatter plot of the blood glucose level of all subjects (at the time of admission, minimum, maximum values during hospitalization, and at discharge, respectively).

**Additional file 3: Fig. S2.** Scatter plots of the blood glucose level of the subjects (at the time of admission, minimum, maximum values during hospitalization, and at discharge, respectively) according to the presence of diabetes were presented. The plot on the left is for diabetic patients, and the plot on the right is for non-diabetic patients.

**Additional file 4: Fig. S3.** Scatter plots of the blood glucose level of the subjects (at the time of admission, minimum, maximum values during hospitalization, and at discharge, respectively) according to the use of insulin during hospitalization were presented. The plot on the left is a plot of patients who used insulin, and the plot on the right is a plot of patients who did not use insulin.

**Additional file 5: Fig. S4.** Spline curves for the hazard ratio of 1-year all-cause mortality according to glucose variability. Glycemic variability was presented by (A) standard deviation (SD) of serum glucose levels and (B) coefficient of variation (CoV) of serum glucose levels.

## Data Availability

The data of this study may be available on reasonable request to the Korean Acute Heart Failure.
