# Supplementary material for: In-hospital glycemic variability and all-cause mortality among patients hospitalized for acute heart failure
Source: Cardiovasc Diabetol. 2022 Dec 27;21:291. doi: 10.1186/s12933-022-01720-4 (PMC9795600; doi:10.1186/s12933-022-01720-4)
Supplement: Supplementary file 1 — Additional file 1: Additional Tables. [file 12933_2022_1720_MOESM1_ESM.docx]

**ADDITIONAL MATERIALS**

Table S1. Baseline characteristics of the study population and excluded patients

|  | Study population  n = 2,617 | Excluded  n = 2,738 | p Value |
| --- | --- | --- | --- |
| Age, years | 72 (61–79) | 71 (60–78) | 0.003 |
| Male sex, n (%) | 1,379 (53) | 1,456 (53) | 0.744 |
| BMI, kg/m^2^ | 23.0 (20.5–25.4) | 23.1 (20.9–25.6) | 0.014 |
| *Past medical history, n (%)* | | | |
| Hypertension | 1,557 (60) | 1,605 (59) | 0.533 |
| Diabetes | 1,110 (42) | 954 (35) | <0.001 |
| CKD | 425 (16) | 324 (12) | <0.001 |
| Previous HF admission | 877 (34) | 835 (31) | 0.020 |
| Ischemic heart disease | 737 (28) | 749 (27) | 0.546 |
| Atrial fibrillation | 906 (35) | 972 (36) | 0.518 |
| ICD | 28 (1.1) | 49 (1.8) | 0.036 |
| CRT | 20 (0.8) | 10 (0.4) | 0.076 |
| *Physical examination at admission* | | | |
| SBP, mmHg | 130 (110–150) | 130 (110–150) | 0.239 |
| DBP, mmHg | 77 (65–89) | 79 (68–90) | <0.001 |
| Pulse rate, beats per min | 91 (76–110) | 88 (72–106) | <0.001 |
| NYHA III/IV, n (%) | 2,290 (88) | 2,228 (81) | <0.001 |
| *Echocardiographic parameters* | | | |
| LVEF, % | 36 (26–51) | 35 (25–48) | <0.001 |
| LVEF ≤40%, n (%) | 1,385 (58) | 1,556 (62) | 0.026 |
| *Laboratory parameters* | | | |
| Serum creatinine, mg/dL | 1.1 (0.8–1.6) | 1.0 (0.8–1.4) | <0.001 |
| Serum sodium, mEq/dL | 138 (135–141) | 139 (136–141) | <0.001 |
| Hemoglobin, g/dL | 12.2 (10.6–13.8) | 12.7 (11.1–14.2) | <0.001 |
| HbA1c, % [mmol/mol], (n = 2,064) | 6.4 (5.8–7.3) [46 (40–56)] | 6.3 (5.8–7.4) [45 (40–57)] | 0.743 |
| BNP, pg/mL, (n = 2,245) | 964 (501–1,860) | 786 (439–1499) | <0.001 |
| NT-proBNP, pg/mL, (n = 3,021) | 5,314 (2,373–13,200) | 4,380 (1,934–10,017) | <0.001 |
| *Treatment during hospitalization, n (%)* | | | |
| Insulin treatment during hospitalization | 805 (31) | 354 (13) | <0.001 |
| Oral hypoglycemic agent use on admission | 661 (25) | 629 (23) | 0.055 |
| Sulfonylurea | 399 (15) | 244 (9) | <0.001 |
| Metformin | 388 (15) | 365 (13) | 0.125 |
| Thiazolidinedione | 22 (0.8) | 17 (0.6) | 0.433 |
| Alpha glucosidase inhibitor | 87 (3) | 87 (3) | 0.821 |
| DPP-4 inhibitor | 138 (5) | 112 (4) | 0.047 |
| Inotropic use | 2361 (90) | 2117 (77) | <0.001 |
| Mechanical ventilation | 556 (21) | 132 (5) | <0.001 |
| RRT | 209 (8) | 88 (3) | <0.001 |
| CRRT | 117 (56) | 26 (30) | <0.001 |
| MCSD | 165 (6) | 31 (1) | <0.001 |
| IABP | 118 (5) | 25 (1) | <0.001 |
| ECMO | 75 (2.9) | 12 (0.4) | <0.001 |
| Duration of hospitalization, days | 10 (7–19) | 7 (4–10) | <0.001 |
| *Medications at discharge, n (%)* | | | |
| ACEi/ARBs | 1,696 (65) | 1,989 (73) | <0.001 |
| Loop diuretics | 1,830 (70) | 2,065 (75) | <0.001 |
| Beta-blockers | 1,232 (47) | 1,561 (57) | <0.001 |
| MRAs | 1,190 (46) | 1,303 (48) | 0.127 |

Values are median (interquartile range), number (%), or mean ± standard deviation.

ACEi/ARB = angiotensin-converting enzyme inhibitor/angiotensin II receptor blocker; BNP = brain natriuretic peptide; CRRT = continuous renal replacement therapy; CRT = cardiac resynchronization therapy; DBP = diastolic blood pressure; ECMO = extracorporeal membrane oxygenation therapy; eGFR = estimated glomerular filtration rate; HF = heart failure; IABP = intra-aortic balloon pump; ICD = implantable cardioverter-defibrillator; LVEF = left ventricular ejection fraction; MCSD = mechanical circulatory support devices; MRA = mineralocorticoid receptor antagonist; NT-proBNP = N-terminal prohormone of brain natriuretic peptide; RRT = renal replacement therapy; SBP = systolic blood pressure.

Table S2. Clinical events during the follow-up period

| Event cases, n (%) | Total, n = 2,617 |
| --- | --- |
| HF readmission | 576 (22) |
| 6-month all-cause mortality | 398 (15) |
| 1-year all-cause mortality | 583 (22) |
| Non-diabetes, n = 1,517 (58% of total population) | |
| HF readmission | 292 (19) |
| 6-month all-cause mortality | 224 (15) |
| 1-year all-cause mortality | 313 (21) |
| Diabetes, n = 1,100 (42% of total population) | |
| HF readmission | 284 (26) |
| 6-month all-cause mortality | 174 (16) |
| 1-year all-cause mortality | 270 (25) |

HF = heart failure.

Table S3. Parameters of glucose level according to the 1-year all-cause mortality.

|  | Event (-)  (n = 2,034) | Event (+)  (n = 583) | Total  n = 2,617 | p Value |
| --- | --- | --- | --- | --- |
| *Glucose parameters* | | | | |
| Mean glucose level, mg/dL | 125 (107–162) | 133 (113–166) | 127 (107–163) | <0.001 |
| Difference of glucose level^*^, mg/dL | 70 (33–137) | 87 (49–144) | 75 (37–140) | <0.001 |
| SD of glucose level, mg/dL | 34.6 (16.9–67.3) | 42.0 (24.8–71.9) | 36.4 (18.2–68.3) | <0.001 |
| CoV of glucose level, % | 27.5 (15.2–43.6) | 31.0 (20.5–46.5) | 28.5 (16.1–43.9) | <0.001 |
| HbA_1c_, % [mmol/mol], (n = 1,039) | 6.4 (5.8–7.3) [46 (40–56)] | 6.6 (5.9 –7.3) [49 (41–56)] | 6.4 (5.8–7.3) [46 (40–56)] | 0.155 |
| *Treatment for diabetes, n (%)* | | | | |
| Insulin treatment during hospitalization | 615 (30) | 190 (33) | 805 (31) | 0.301 |
| Oral hypoglycemic agents use on admission | 403 (20) | 105 (18) | 508 (19) | 0.362 |
| Sulfonylurea | 314 (15) | 85 (15) | 399 (15) | 0.658 |
| Metformin | 320 (16) | 68 (12) | 388 (15) | 0.018 |
| Thiazolidinedione | 13 (1) | 9 (2) | 22 (1) | 0.064 |
| Alpha glucosidase inhibitor | 67 (3) | 20 (3) | 87 (3) | 0.975 |
| DPP-4 inhibitor | 109 (5) | 29 (5) | 138 (5) | 0.794 |

Values are median (interquartile range) or number (%).

CoV = coefficient of variation; DPP-4 = dipeptidyl peptidase-4; SD = standard deviation.

^*^Difference of glucose level: maximal value – minimal value of measured glucose level

Table S4. Cutoff of glucose variability parameters according to the 1-year all-cause mortality (Youden method)

|  | Total | Non-diabetes | Diabetes |
| --- | --- | --- | --- |
| SD of glucose level, mg/dL | 24.5 | 24.5 | 68.3 |
| Specificity | 0.374 | 0.508 | 0.430 |
| Sensitivity | 0.762 | 0.684 | 0.600 |
| CoV of glucose level, % | 20.9 | 20.5 | 21.8 |
| Specificity | 0.379 | 0.472 | 0.239 |
| Sensitivity | 0.744 | 0.703 | 0.811 |

CoV = coefficient of variation; SD = standard deviation

Table S5. Baseline characteristics according to glycemic variability (cutoff of CoV = 21%)

|  | Low GV  (CoV ≤21%)  n = 924 | High GV  (CoV >21%)  n = 1,693 | p Value |
| --- | --- | --- | --- |
| Age, years | 72 (61–79) | 72 (61–79) | 0.887 |
| Male sex | 480 (52) | 899 (53) | 0.601 |
| BMI, kg/m^2^ | 23.1 (20.8–25.7) | 22.8 (20.4–25.2) | 0.02 |
| *Past medical history, n (%)* | | | |
| Hypertension | 532 (58) | 1,025 (61) | 0.151 |
| Diabetes | 241 (26) | 869 (51) | <0.001 |
| CKD | 103 (11) | 322 (19) | <0.001 |
| Previous HF admission | 288 (31) | 589 (35) | 0.067 |
| Ischemic heart disease | 229 (25) | 508 (30) | 0.005 |
| Atrial fibrillation | 372 (40) | 534 (32) | <0.001 |
| ICD | 10 (1.1) | 18 (1.1) | >0.999 |
| CRT | 4 (0.4) | 16 (0.9) | 0.229 |
| *Physical examination at admission* | | | |
| SBP, mmHg | 129 (110–150) | 130 (109–150) | 0.903 |
| DBP, mmHg | 78.0 (67–90) | 76.0 (64–88) | 0.008 |
| Pulse rate, beats/min | 89.5 (74–107) | 93.0 (78–111) | <0.001 |
| NYHA III/IV, n (%) | 791 (86) | 1499 (89) | 0.035 |
| *Echocardiographic parameters* | | | |
| LVEF, % | 38.0 (26.8–53.0) | 35.3 (26.0–50.0) | 0.018 |
| LVEF ≤40, n (%) | 463 (54) | 922 (61) | 0.004 |
| *Laboratory parameter* | | | |
| Serum creatinine, mg/dL | 1.0 (0.8–1.4) | 1.2 (0.9–1.7) | <0.001 |
| Serum sodium, mEq/L | 139 (136–141) | 138 (134–140) | <0.001 |
| Hemoglobin, g/dL | 12.5 ± 2.2 | 12.1 ± 2.3 | <0.001 |
| HbA1c, % (n = 1,039) | 6.2 (5.8–6.8) | 6.6 (5.9–7.5) | <0.001 |
| BNP, pg/mL (n = 1,224) | 805 (433–1,569) | 1026 (552–2,042) | <0.001 |
| NT-proBNP, pg/mL (n = 1,263) | 3969 (1,897–9,426) | 6265 (2,794–15,503) | <0.001 |
| *Treatment during hospitalization, n (%)* | | | |
| Inotropic use | 789 (85) | 1572 (93) | <0.001 |
| Mechanical ventilation | 67 (7) | 489 (29) | <0.001 |
| RRT | 37 (4) | 172 (10) | <0.001 |
| CRRT | 18 (49) | 74 (43) | 0.658 |
| MCSD | 30 (3) | 135 (8) | <0.001 |
| IABP | 21 (2) | 97 (6) | <0.001 |
| ECMO | 11 (1) | 64 (4) | <0.001 |
| *Medications at discharge, n (%)* | | | |
| ACEi/ARBs | 645 (70) | 1051 (62) | <0.001 |
| Loop diuretics | 704 (76) | 1126 (67) | <0.001 |
| Beta-blockers | 450 (49) | 782 (46) | 0.234 |
| MRAs | 475 (51) | 715 (42) | <0.001 |

Values are median (interquartile range), number (%), or mean ± SD.

Abbreviations as in Tables S1–3.Table S6. Baseline characteristics according to diabetes and glycemic variability (cutoff of CoV = 21%)

|  | No diabetes  (n = 1,507) | | Diabetes  (n = 1,110) | |
| --- | --- | --- | --- | --- |
|  | CoV ≤21%  (n = 683) | CoV >21%  (n = 824) | CoV ≤21%  (n = 241) | CoV >21%  (n = 869) |
| Age, yrs | 73 [59 - 80] | 71 [57 - 80] | 71 [63 - 77] | 72 [63 - 78] |
| Male sex, n (%) | 354 (52) | 420 (51) | 126 (52) | 479 (55) |
| BMI, kg/m^2^ | 22.9 [20.4 - 25.5] | 22.3 [20.0 - 24.8] | 23.8 [21.7 - 26.6] | 23.3 [21.0 - 25.5] |
| Past medical history, n (%) | | | | |
| Hypertension | 353 (52) | 403 (49) | 179 (74) | 622 (72) |
| Diabetes | 0 ( 0) | 0 ( 0) | 241 (100) | 869 (100) |
| CKD | 55 ( 8) | 99 (12) | 48 (20) | 223 (26) |
| Previous HF admission | 202 (30) | 257 (31) | 86 (36) | 332 (38) |
| Ischemic heart disease | 142 (21) | 159 (19) | 87 (36) | 349 (40) |
| Atrial fibrillation | 281 (41) | 299 (36) | 91 (38) | 235 (27) |
| ICD | 7 ( 1.0) | 8 ( 1.0) | 3 ( 1.2) | 10 ( 1.2) |
| CRT | 1 ( 0.1) | 6 ( 0.7) | 3 ( 1.2) | 10 ( 1.2) |
| Physical examination on admission | | | | |
| SBP, mmHg | 128 [110 - 150] | 126 [105 - 147] | 130 [110 - 153] | 132 [111 - 154] |
| DBP, mmHg | 78 [67 - 90] | 75 [64 - 87] | 79 [68 - 90] | 77 [64 - 89] |
| Pulse rate, beats/min | 90 [74 - 107] | 94 [78 - 112] | 89 [75 - 104] | 92 [77 - 111] |
| NYHA III/IV, n (%) | 575 (84) | 728 (88) | 216 (90) | 771 (89) |
| Echocardiographic parameters | | | | |
| LVEF, % (n = 2,542) | 39 [27 - 54] | 38 [26 - 53] | 37 [25 - 49] | 35 [27 - 49] |
| LVEF ≤40%, n (%) | 333 (50) | 428 (53) | 135 (58) | 487 (58) |
| Laboratory parameters | | | | |
| Serum creatinine, mg/dL | 1.0 [ 0.8; 1.3] | 1.1 [ 0.8; 1.5] | 1.1 [ 0.9; 1.7] | 1.3 [ 0.9; 2.0] |
| eGFR, mL/min/1.73m^2^ | 72 [52 - 90] | 64 [42 - 86] | 60 [35 - 81] | 50 [30 - 74] |
| Serum sodium, mEq/L | 139 [136 - 141] | 138 [134 - 141] | 138 [135 - 141] | 137 [134 - 140] |
| Hemoglobin, g/dL | 12.6 ± 2.3 | 12.4 ± 2.3 | 12.1 ± 2.0 | 11.8 ± 2.3 |
| BNP, pg/mL (n = 1,224) | 793 [427 - 1628] | 1017 [524 - 2211] | 881 [437 - 1451] | 1028 [587 - 1860] |
| NT-proBNP, pg/mL (n = 1,263) | 3858 [1780 - 7891] | 5880 [2781 - 14288] | 4131 [1963 - 12538] | 6659 [2820 - 16691] |
| Treatments during index hospitalization, n (%) | | | | |
| Inotropic use | 574 (84) | 770 (93) | 215 (89) | 802 (92) |
| Mechanical ventilation | 46 ( 7) | 260 (32) | 21 ( 9) | 229 (26) |
| RRT | 22 ( 3) | 77 ( 9) | 15 ( 6) | 95 (11) |
| CRRT | 10 (46) | 29 (38) | 8 (53) | 45 (47) |
| MCSD | 16 ( 2.3%) | 73 ( 8.9%) | 14 ( 5.8%) | 62 ( 7.1%) |
| IABP | 10 ( 1.5%) | 51 ( 6.2%) | 11 ( 4.6%) | 46 ( 5.3%) |
| ECMO | 7 ( 1.0%) | 35 ( 4.2%) | 4 ( 1.7%) | 29 ( 3.3%) |
| Duration of hospitalization, days | 8 [5 – 12] | 14 [8 – 26] | 8 [6 – 12] | 12 [7 – 21] |
| Medications at discharge, n (%) | | | | |
| ACEi/ARBs | 485 (71) | 481 (58) | 160 (66) | 570 (66) |
| Loop diuretics | 523 (77) | 535 (65) | 181 (75) | 591 (68) |
| Beta-blockers | 317 (46) | 335 (41) | 133 (55) | 447 (51) |
| MRAs | 350 (51) | 361 (44) | 125 (52) | 354 (41) |
| Treatment for diabetes, n (%) | | | | |
| Insulin treatment during hospitalization | 21 ( 3) | 155 (19) | 95 (39) | 534 (61) |
| Sulfonylurea | 0 ( 0) | 0 ( 0) | 68 (28) | 331 (38) |
| Metformin | 0 ( 0) | 0 ( 0) | 88 (37) | 300 (35) |
| Thiazolidinedione | 0 ( 0) | 0 ( 0) | 5 ( 2) | 17 ( 2) |
| Alpha glucosidase inhibitor | 0 ( 0) | 0 ( 0) | 22 ( 9) | 65 ( 8) |

Values are median (interquartile range), number (%), or mean ± standard deviation.

CoV = coefficient of variation; Other abbreviations as in Table S1.

Table S7. Univariate Cox proportional regression analysis for the 1-year all-cause mortality

|  | HR | 95% confidence interval | | p Value |
| --- | --- | --- | --- | --- |
| Age (per 1 year) | 1.04 | 1.033 | 1.047 | <0.001 |
| Male sex | 0.810 | 0.688 | 0.952 | 0.011 |
| BMI (per 1 kg/m^2^) | 0.922 | 0.901 | 0.944 | <0.001 |
| SBP (per 1 mmHg) | 0.997 | 0.995 | 1.000 | 0.048 |
| DBP (per 1 mmHg) | 0.993 | 0.989 | 0.998 | 0.005 |
| Hemoglobin (per 1 g/dL) | 0.850 | 0.821 | 0.880 | <0.001 |
| eGFR (per 1 mL/min/1.73m^2^) | 0.986 | 0.983 | 0.989 | <0.001 |
| High NP level (yes vs. no)^*^ (n=2,391) | 1.926 | 1.454 | 2.551 | <0.001 |
| *Past medical history* | | | | |
| HTN (yes vs. no) | 1.501 | 1.261 | 1.786 | <0.001 |
| DM (yes vs. no) | 1.163 | 0.989 | 1.369 | 0.069 |
| CKD (yes vs. no) | 1.987 | 1.652 | 2.39 | <0.001 |
| Previous HF admission (yes vs. no) | 1.449 | 1.229 | 1.708 | <0.001 |
| Ischemic heart disease (yes vs. no) | 1.284 | 1.08 | 1.525 | 0.005 |
| Atrial fibrillation (yes vs. no) | 1.124 | 0.951 | 1.33 | 0.171 |
| ICD (yes vs. no) | 1.225 | 0.610 | 2.462 | 0.568 |
| CRT (yes vs. no) | 1.239 | 0.554 | 2.769 | 0.602 |
| *Echocardiographic parameters* | | | | |
| LVEF (per 1% increase) (n = 2,542) | 0.998 | 0.993 | 1.004 | 0.579 |
| LVEF <40% (yes vs. no) | 0.987 | 0.828 | 1.176 | 0.88 |
| *Treatment during hospitalization* | | | | |
| Insulin treatment during hospitalization (yes vs. no) | 1.081 | 0.909 | 1.286 | 0.377 |
| Oral hypoglycemic agent use on admission (yes vs. no) | 0.875 | 0.709 | 1.081 | 0.217 |
| Sulfonylurea (yes vs. no) | 0.907 | 0.720 | 1.141 | 0.404 |
| Inotropic use (yes vs. no) | 1.431 | 1.046 | 1.959 | 0.025 |
| RRT (yes vs. no) | 2.11 | 1.662 | 2.679 | <0.001 |
| MCSD (yes vs. no) | 0.663 | 0.459 | 0.958 | 0.055 |
| Duration of hospitalization (per 1 day) | 1.007 | 1.004 | 1.01 | <0.001 |
| *Medications at discharge* | | | | |
| ACEi/ARBs (yes vs. no) | 0.645 | 0.548 | 0.759 | <0.001 |
| Loop diuretics (yes vs. no) | 1.007 | 0.843 | 1.204 | 0.936 |
| Beta-blockers (yes vs. no) | 0.640 | 0.542 | 0.757 | <0.001 |
| MRAs (yes vs. no) | 0.807 | 0.685 | 0.952 | 0.011 |
| *Glucose parameter* | | | | |
| CoV >21% (yes vs. no) | 1.66 | 1.38 | 1.998 | <0.001 |

HRs are expressed as “vs.” for dichotomous variables and per unit for continuous variables. Men coded as 1 and women as 0. Yes coded as 1 and no as 0.

Abbreviations as in Tables S1–3.

^*^Data analyzed with high natriuretic peptide (BNP ≥500 pg/ml or NT-proBNP ≥1000 pg/ml) levels at admission, in available subjects (n = 2,391).

Table S8. Descriptive statistics according to insulin treatment during hospitalization.

|  | No insulin (n = 1,812) | Insulin (n = 805) | P-value |
| --- | --- | --- | --- |
| Duration of hospitalization (days) | 9 [6 – 15] | 16 [9 – 29] | <0.001 |
| Analyzed number of glucose measurements | | | <0.001 |
| 3 times | 1,380 (76%) | 501 (62%) |  |
| 4 times | 432 (24%) | 304 (38%) |  |
| SD of glucose level, mg/dL | 27.8 [14.5 – 48.2] | 70.3 [39.0 – 107.3] | <0.001 |
| CoV of glucose level, % | 23.2 [13.3 – 35.6] | 43.6 [28.9 – 59.7] | <0.001 |

Values are median [interquartile range] or number (%).

SD = standard deviation; CoV = coefficient of variation.
