# Supplementary material for: In-hospital glycemic variability and all-cause mortality among patients hospitalized for acute heart failure
Source: Cardiovasc Diabetol. 2022 Dec 27;21:291. doi: 10.1186/s12933-022-01720-4 (PMC9795600; doi:10.1186/s12933-022-01720-4)
Supplement: Supplementary file 2 — Additional file 2: Fig. S1. Scatter plot of the blood glucose level of all subjects (at the time of admission, minimum, maximum values during hospitalization, and at discharge, respectively). [file 12933_2022_1720_MOESM2_ESM.pdf]

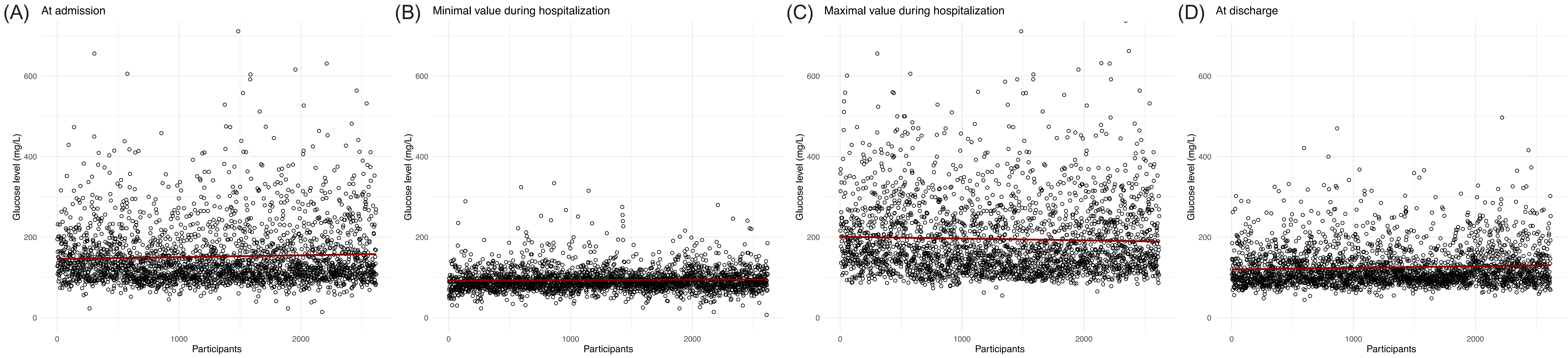
(A)
At admission
(B)
Minimal value during hospitalization
(C)
Maximal value during hospitalization
(D)
At discharge
Glucose level (mg/L)
Participants
0
200
400
600
0
1000
2000
