# Supplementary figures and images for: In-hospital glycemic variability and all-cause mortality among patients hospitalized for acute heart failure
Source: Cardiovasc Diabetol. 2022 Dec 27;21:291. doi: 10.1186/s12933-022-01720-4 (PMC9795600; doi:10.1186/s12933-022-01720-4)

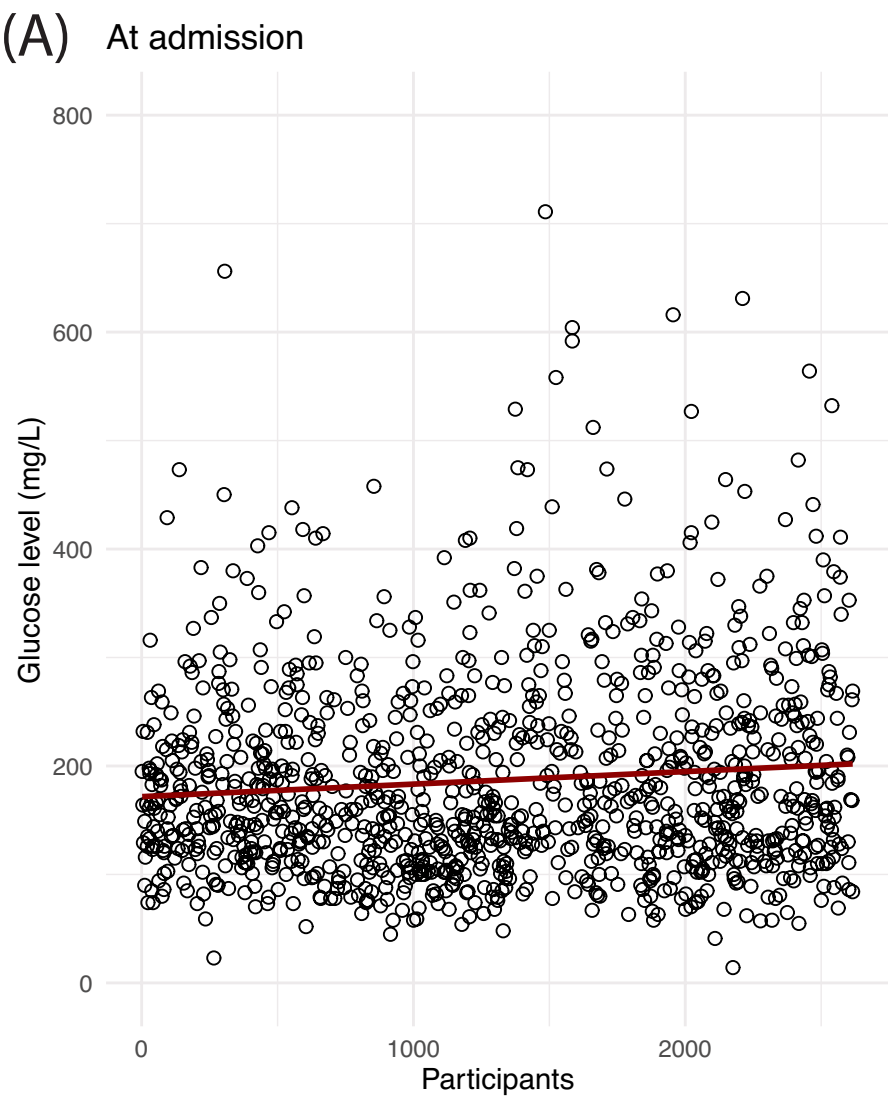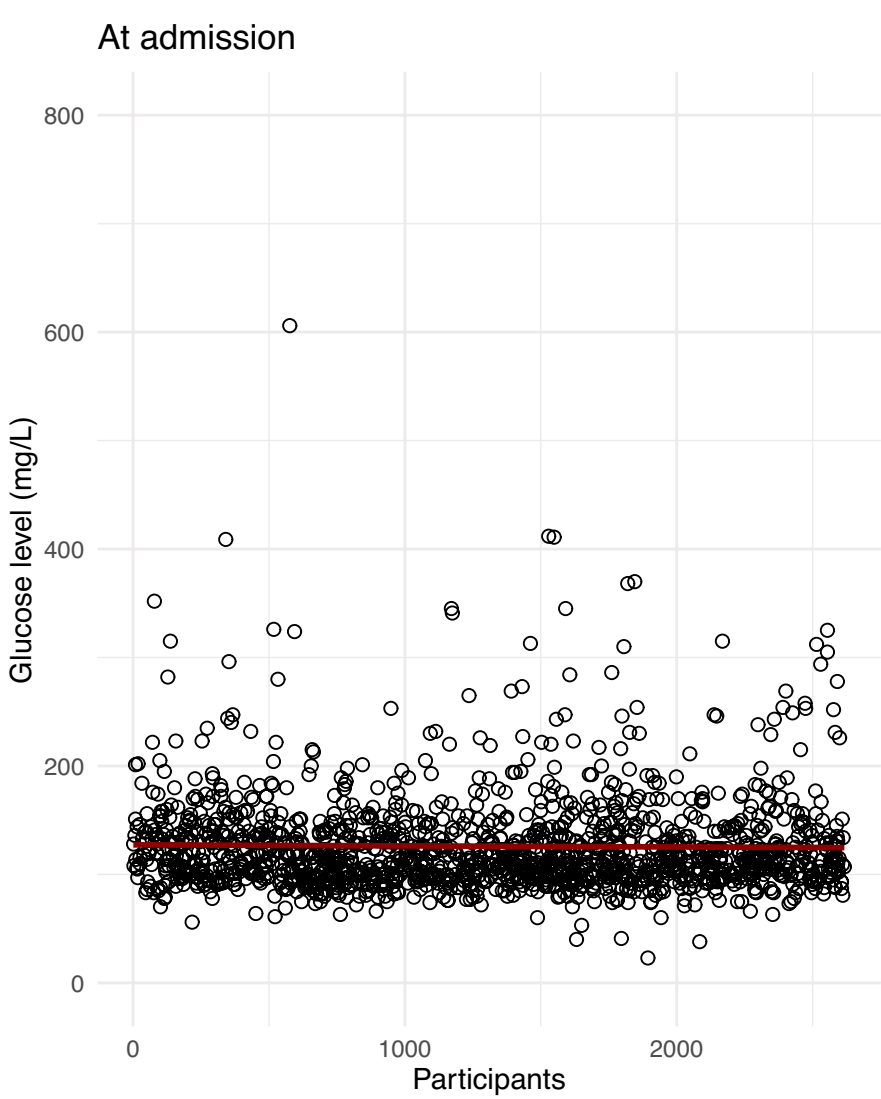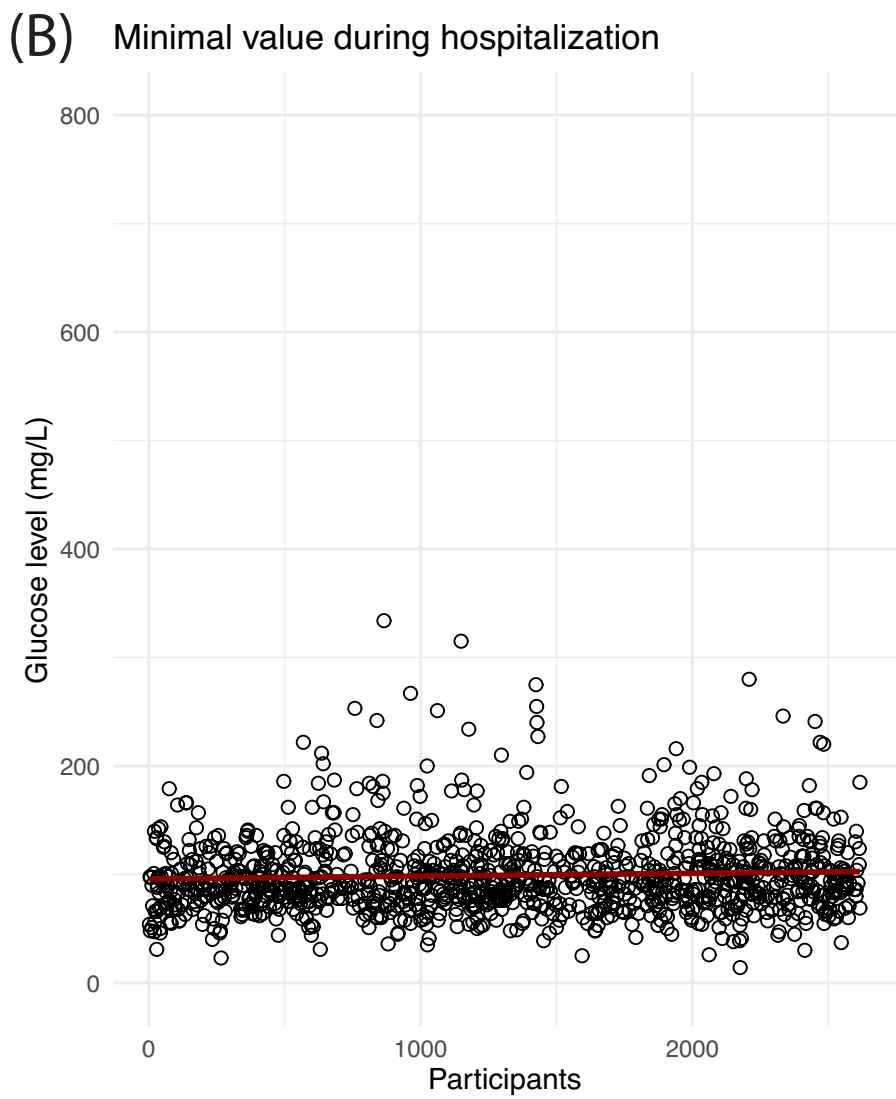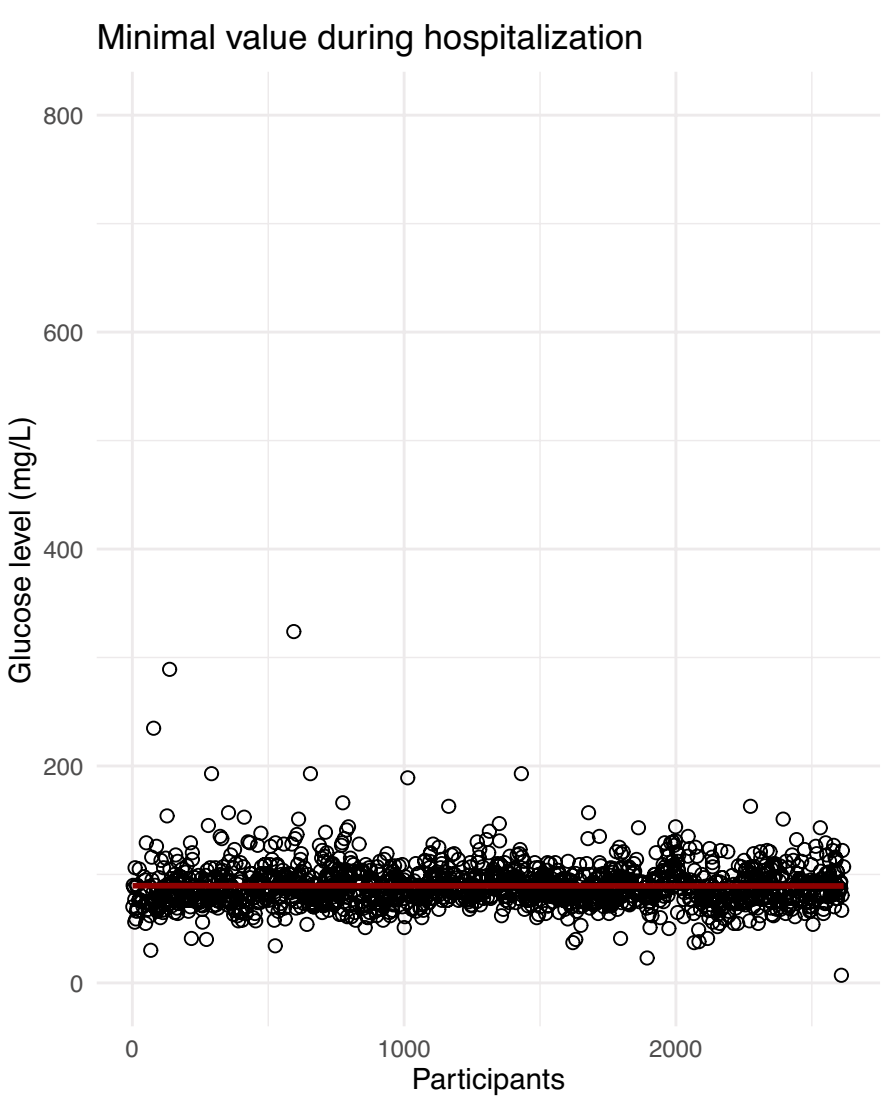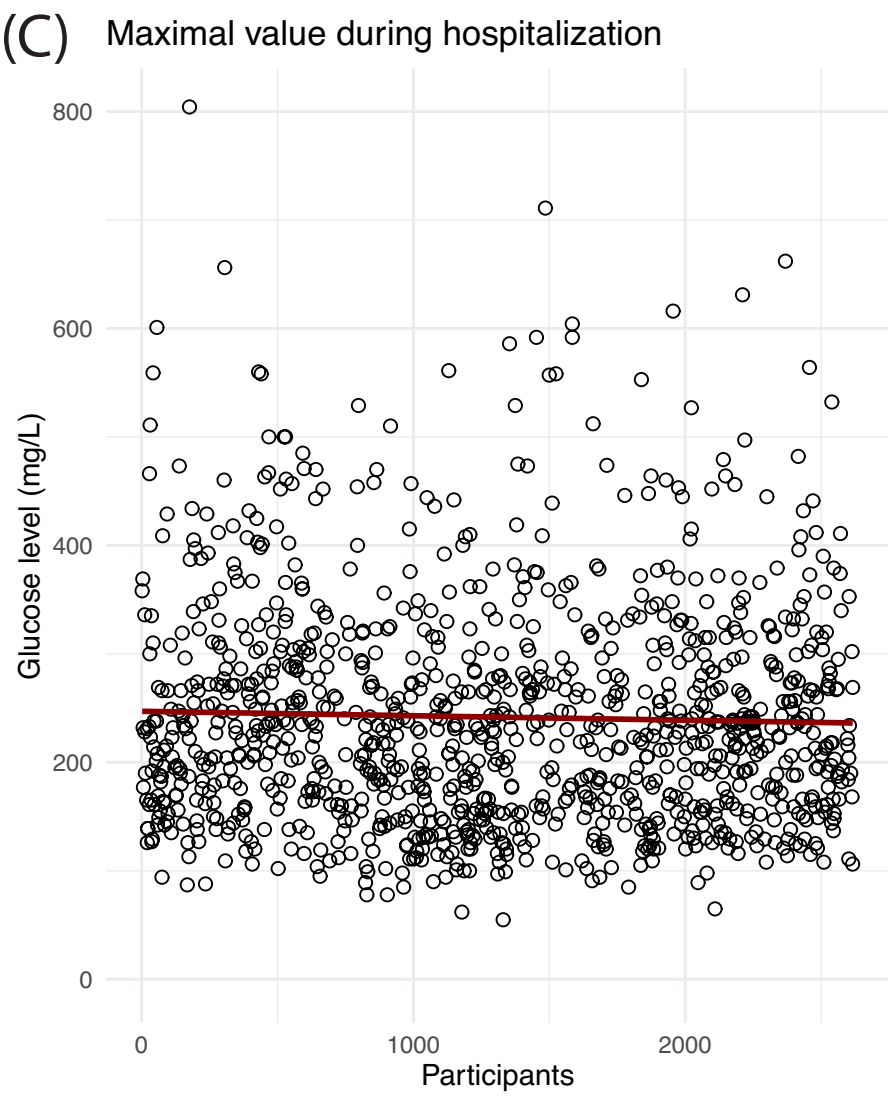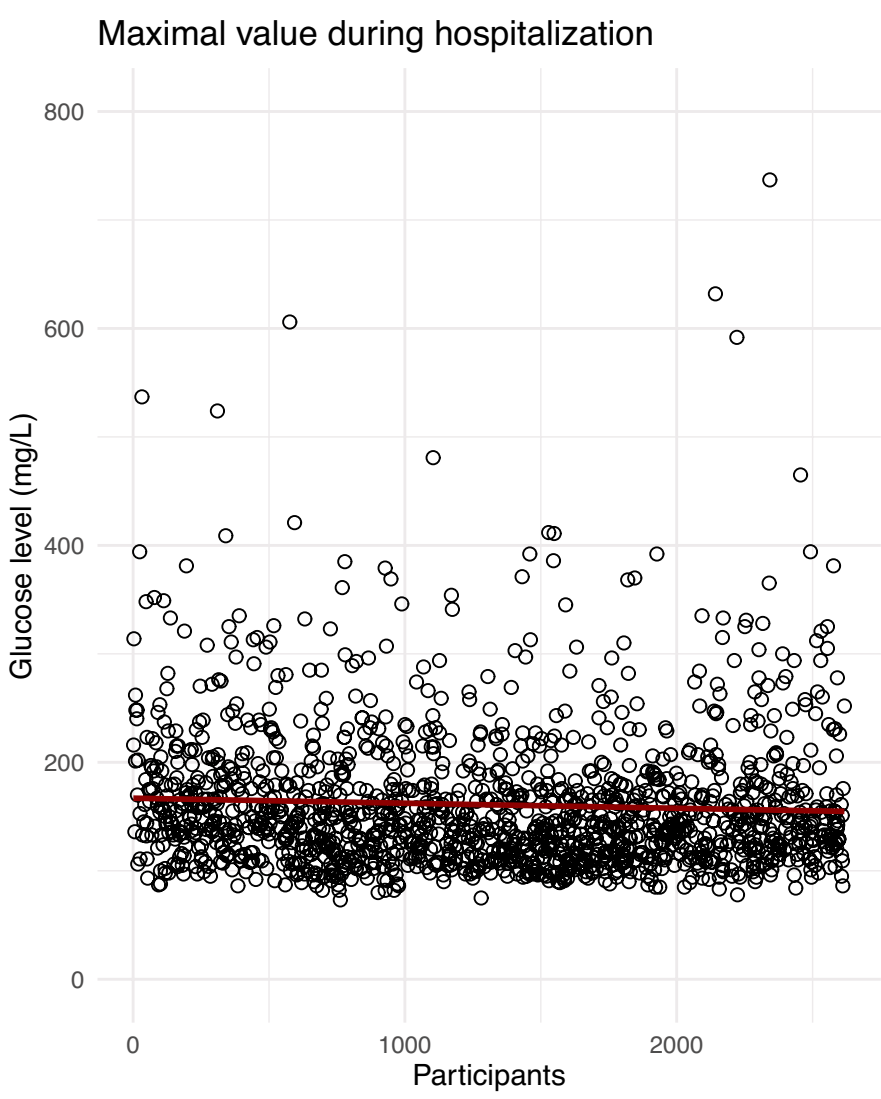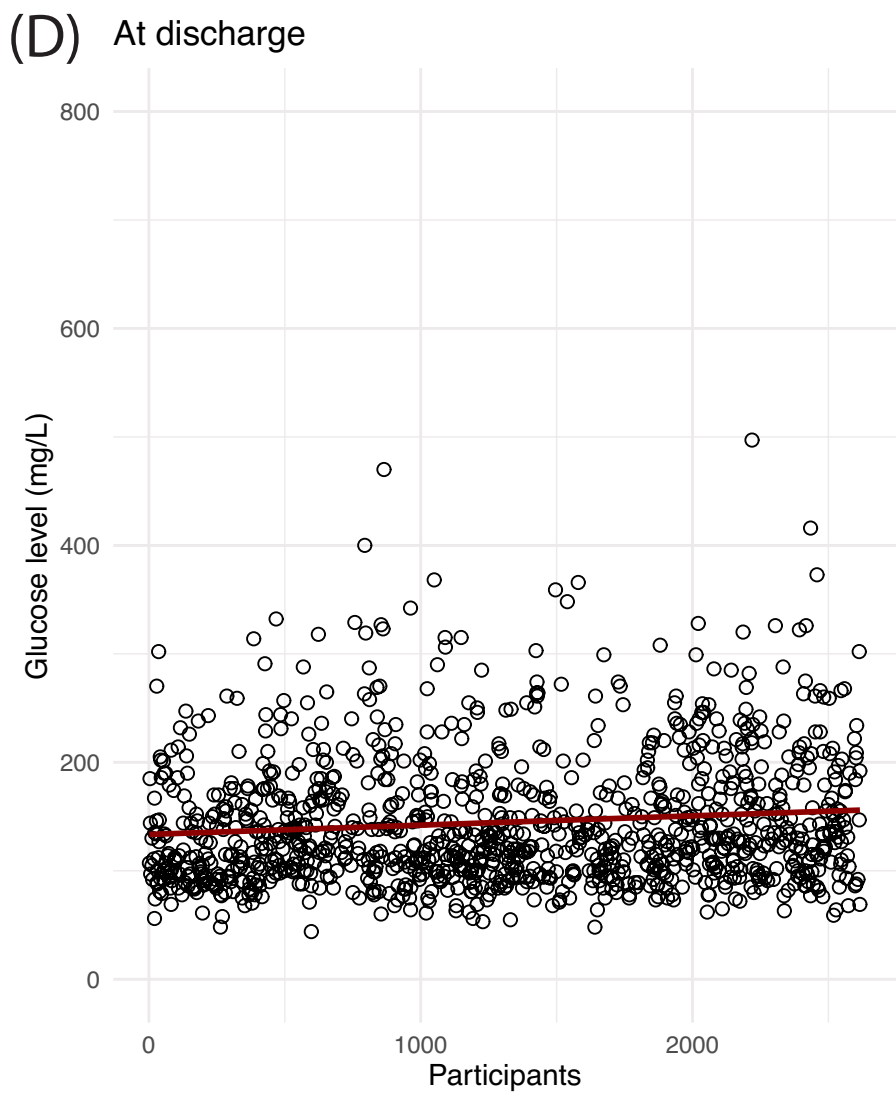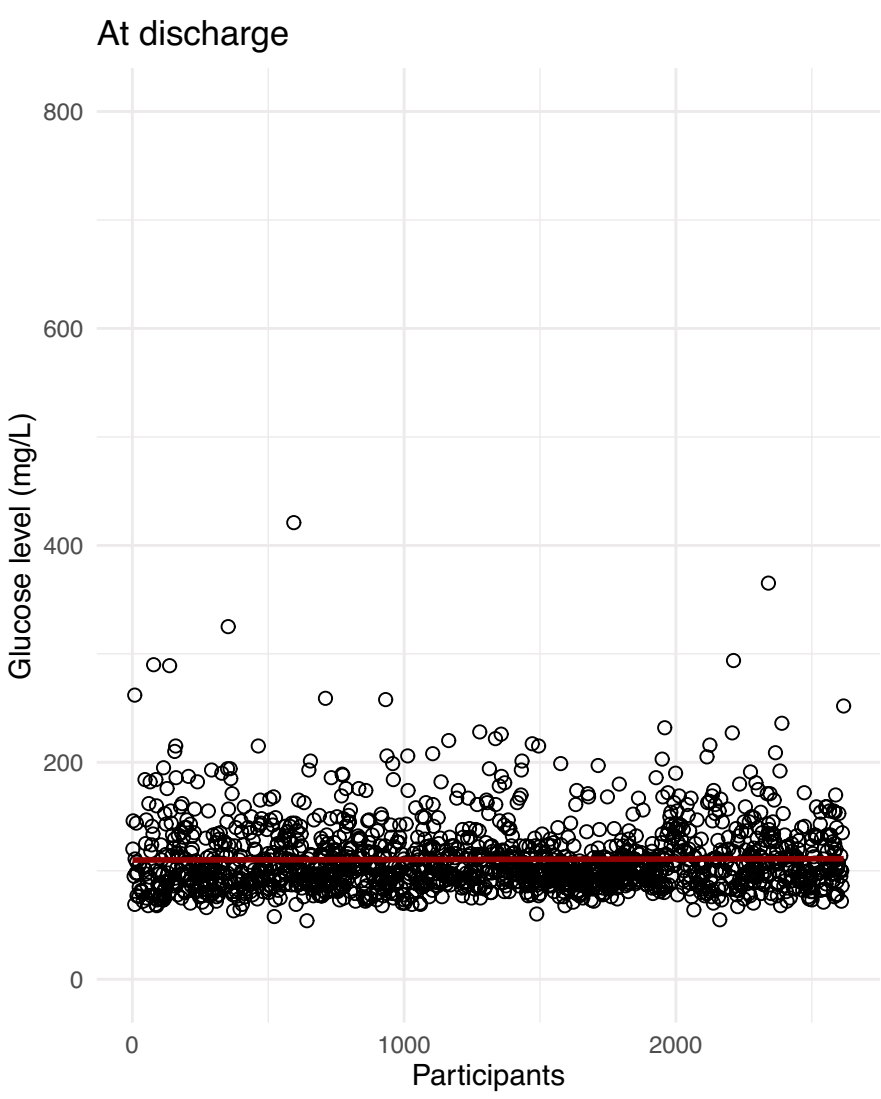

Supplement: Supplementary file 3 — Additional file 3: Fig. S2. Scatter plots of the blood glucose level of the subjects (at the time of admission, minimum, maximum values during hospitalization, and at discharge, respectively) according to the presence of diabetes were presented. The plot on the left is for diabetic patients, and the plot on the right is for non-diabetic patients. [file 12933_2022_1720_MOESM3_ESM.pdf]

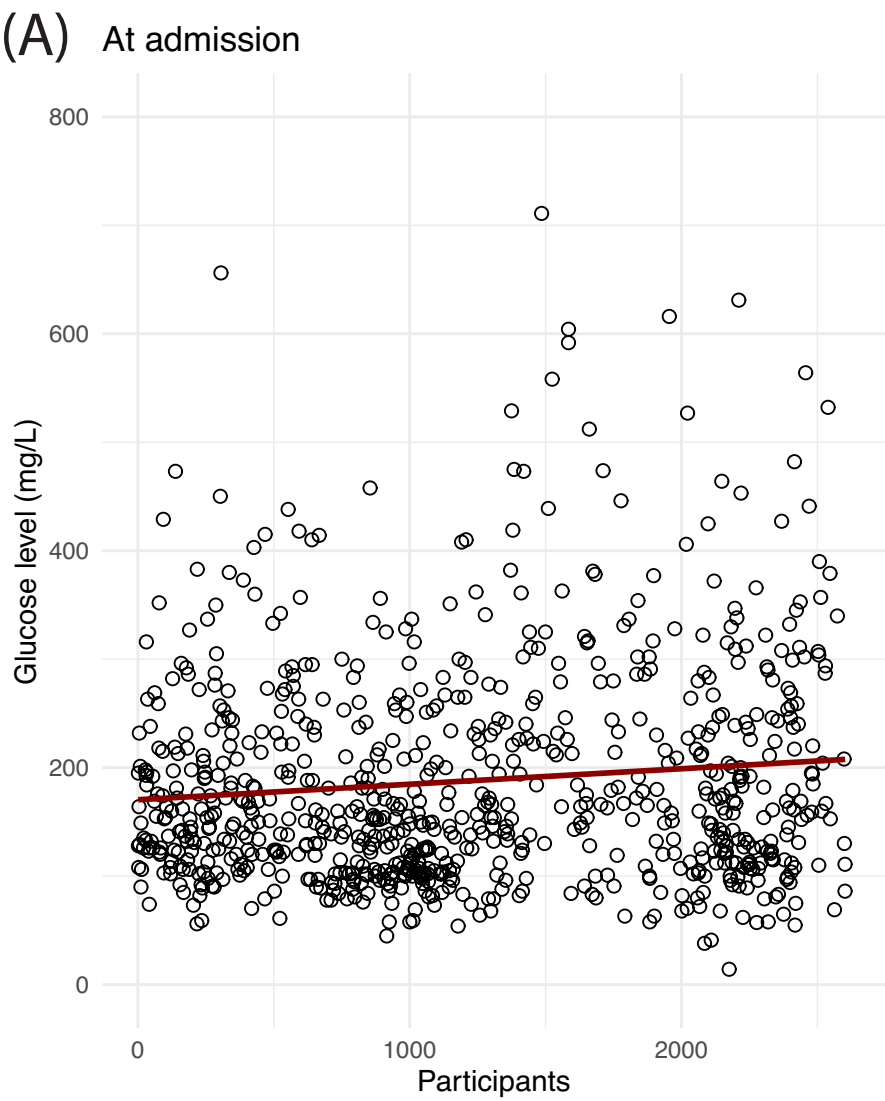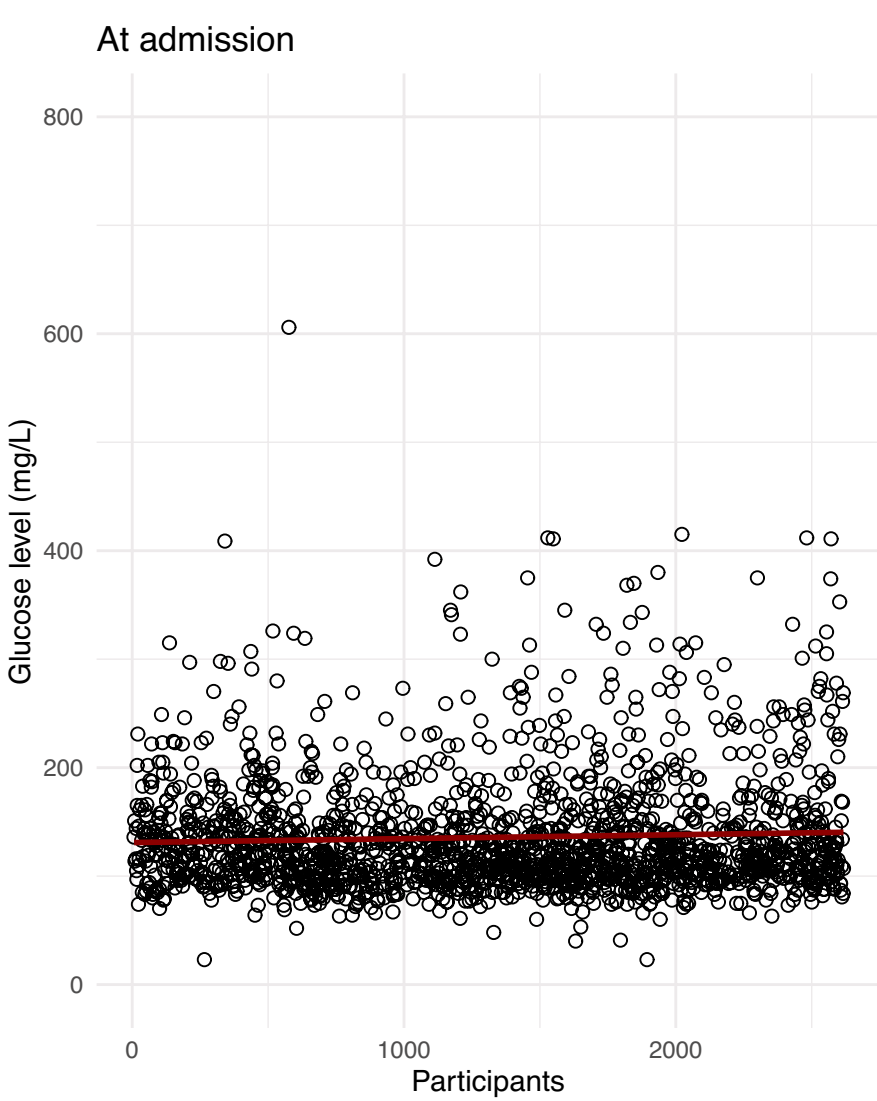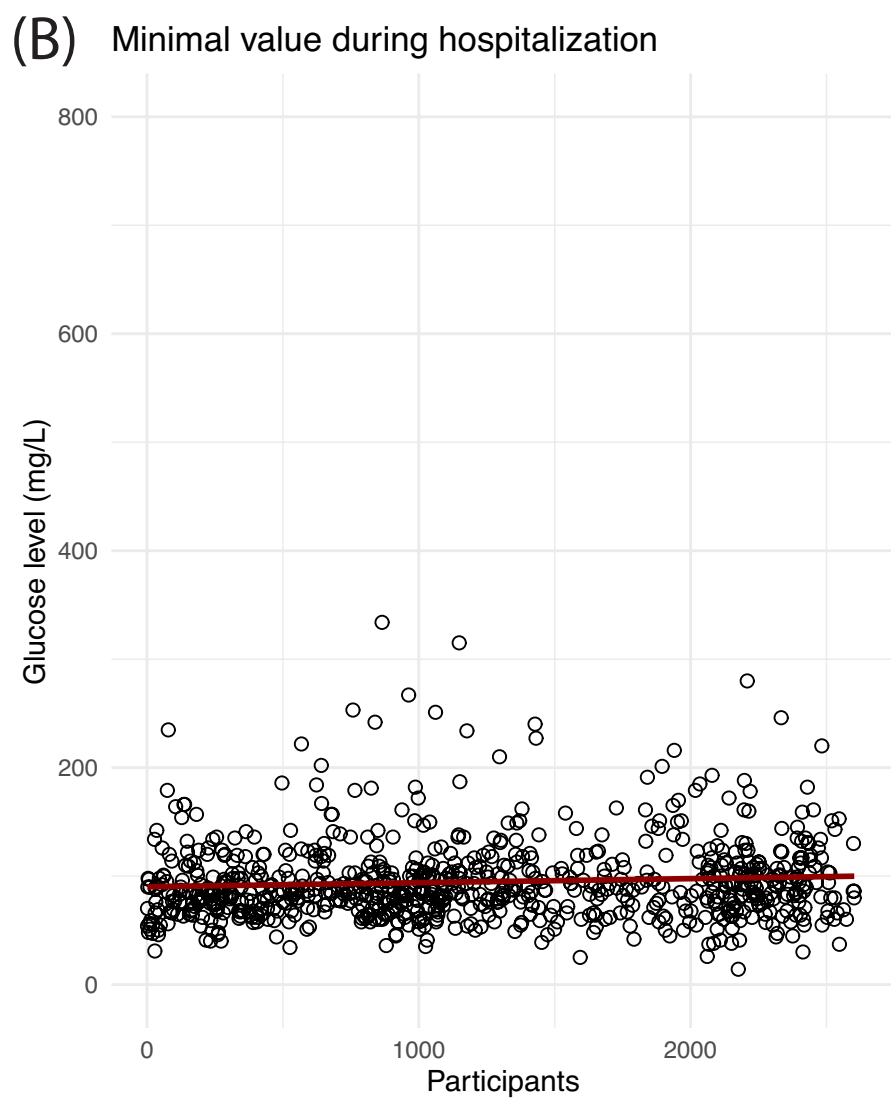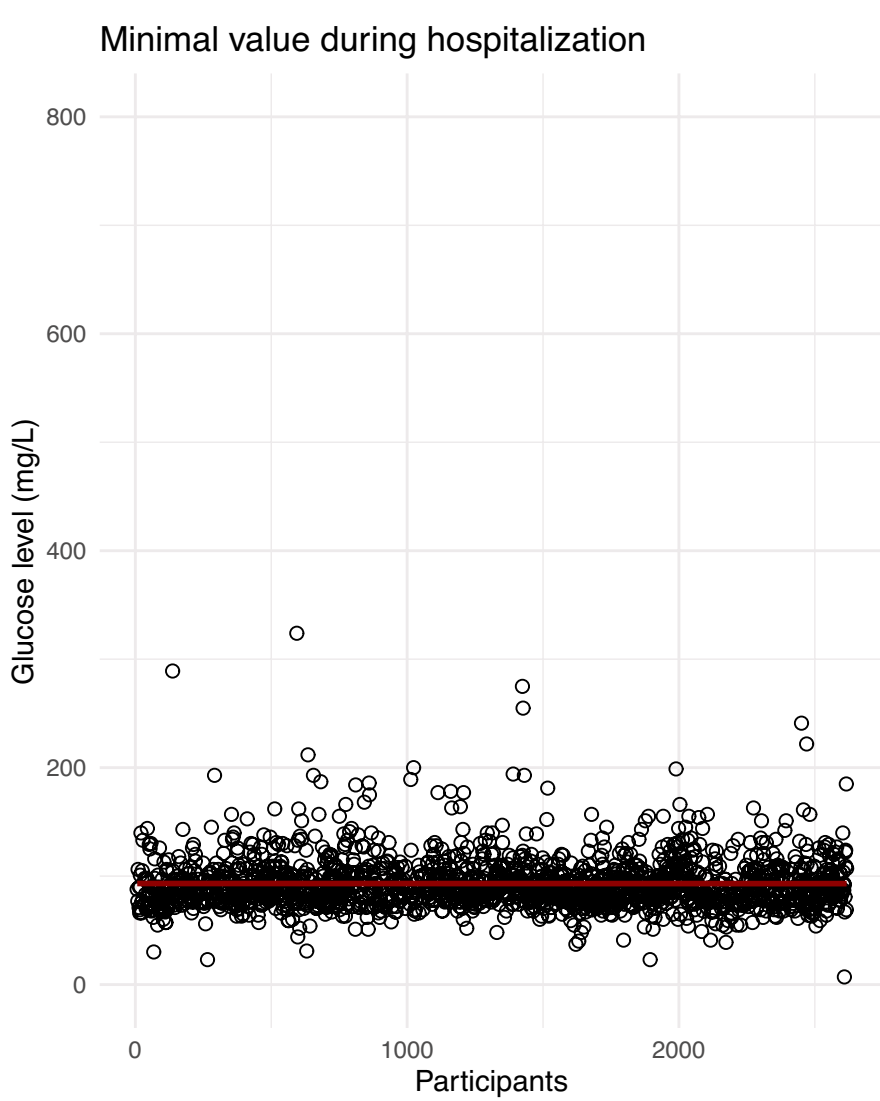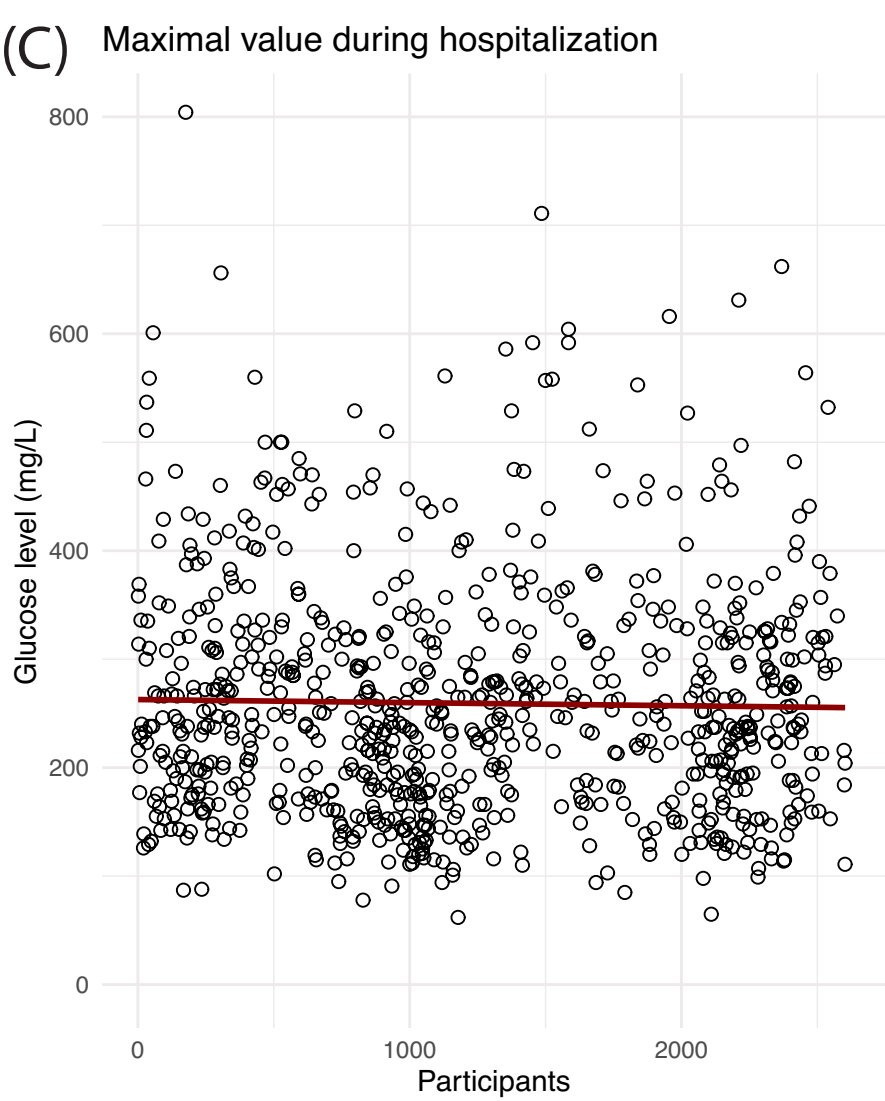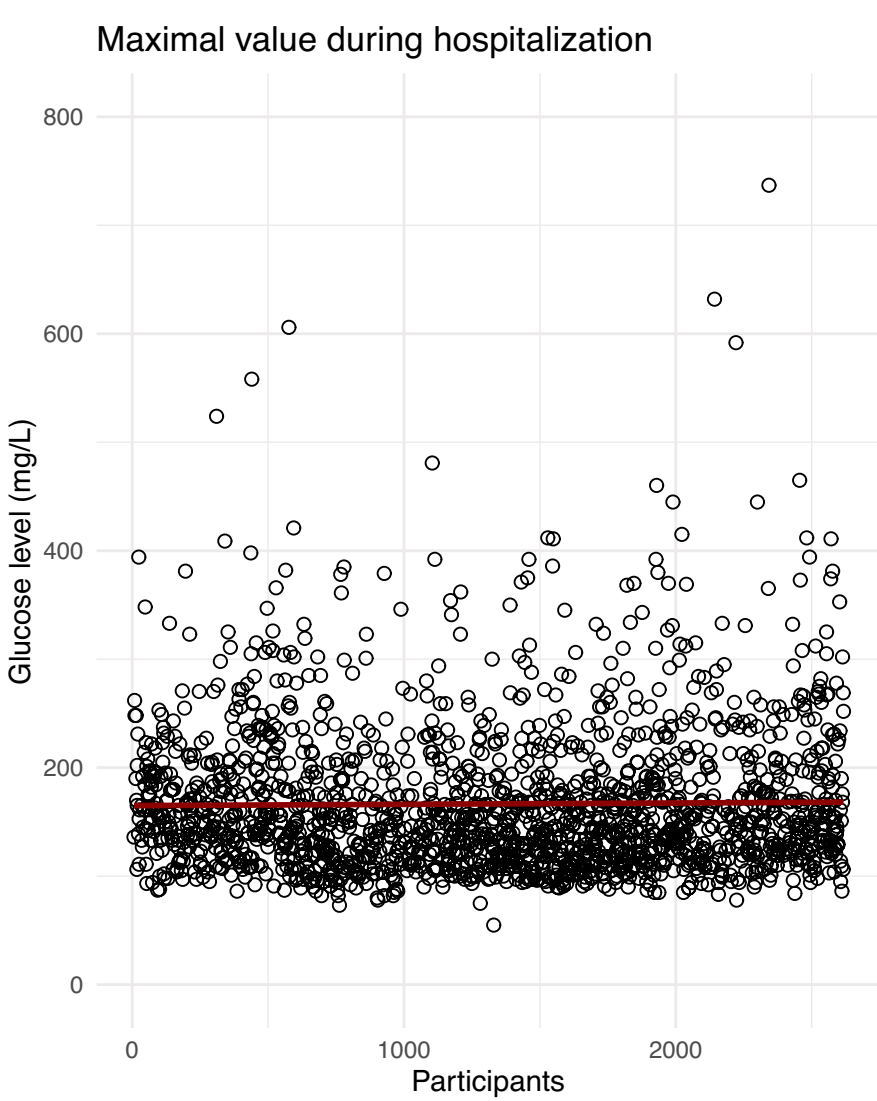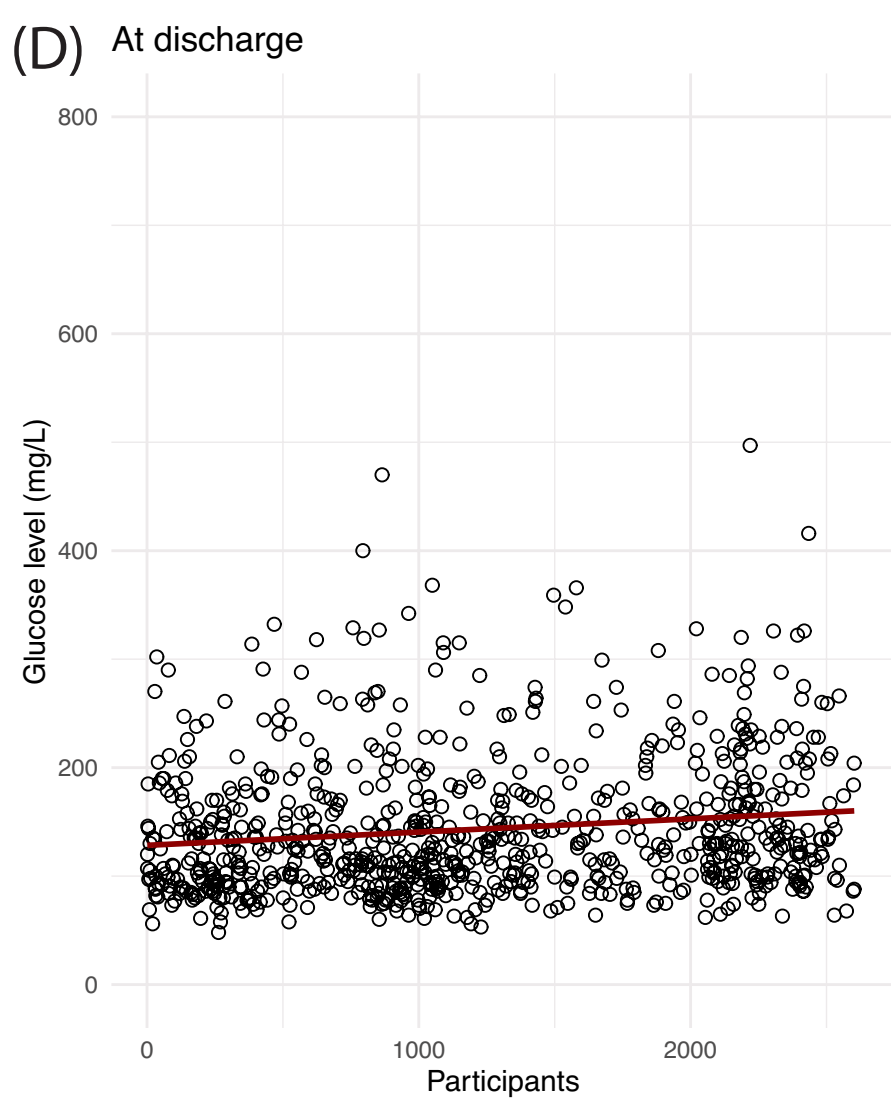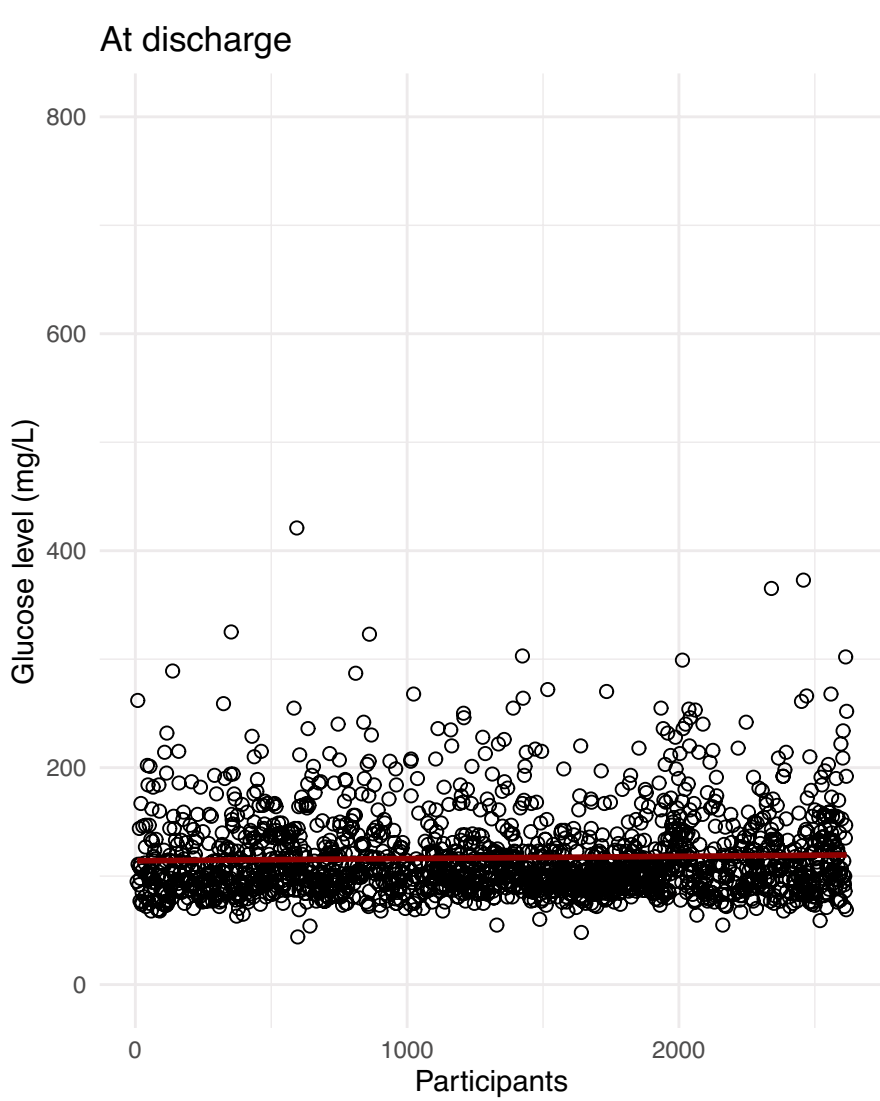

Supplement: Supplementary file 4 — Additional file 4: Fig. S3. Scatter plots of the blood glucose level of the subjects (at the time of admission, minimum, maximum values during hospitalization, and at discharge, respectively) according to the use of insulin during hospitalization were presented. The plot on the left is a plot of patients who used insulin, and the plot on the right is a plot of patients who did not use insulin. [file 12933_2022_1720_MOESM4_ESM.pdf]

(A)

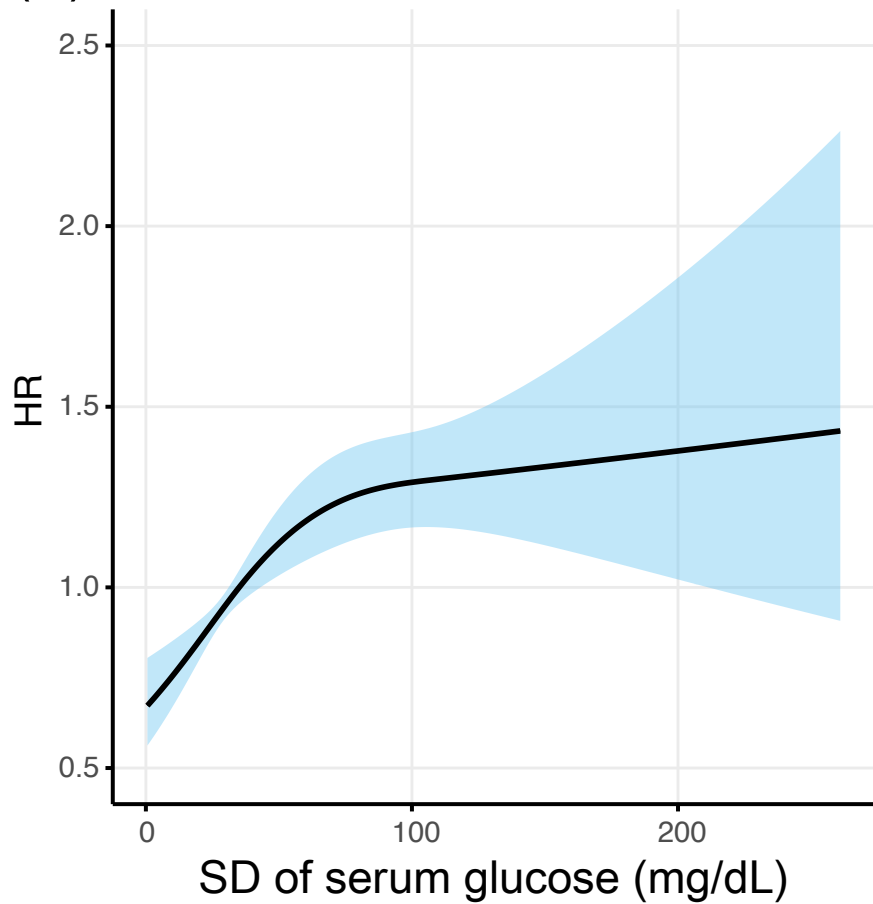

(B)

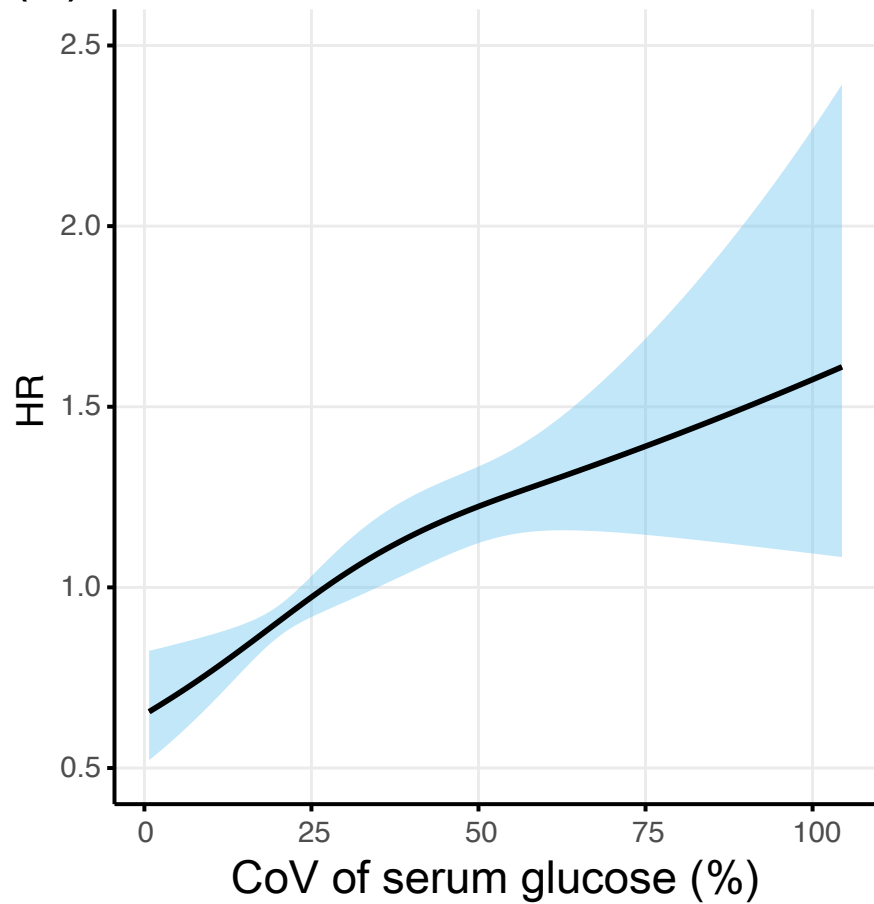

Supplement: Supplementary file 5 — Additional file 5: Fig. S4. Spline curves for the hazard ratio of 1-year all-cause mortality according to glucose variability. Glycemic variability was presented by (A) standard deviation (SD) of serum glucose levels and (B) coefficient of variation (CoV) of serum glucose levels. [file 12933_2022_1720_MOESM5_ESM.pdf]
